# Formation and structure of the first metal complexes comprising amidino­guanidinate ligands

**DOI:** 10.1107/S2056989016015322

**Published:** 2016-10-04

**Authors:** Farid M. Sroor, Phil Liebing, Cristian G. Hrib, Daniel Gräsing, Liane Hilfert, Frank T. Edelmann

**Affiliations:** a Organometallic and Organometalloid Chemistry Department, National Research, Centre, 12622 Dokki, Cairo, Egypt; b Chemisches Institut der Otto-von-Guericke-Universität Magdeburg, Universitätsplatz 2, 39106 Magdeburg, Germany

**Keywords:** crystal structure, amidinate ligands, guanidinate ligands, amidino­guanidinate, lithium, holmium

## Abstract

The first metal complexes comprising amidino­guanidinate ligands have been prepared and structurally characterized, namely bis­[μ-*N*,*N*′,*N*′′,*N*′′′-tetraisopropyl-1-(1-butyl­amidinato)guanidinato-κ^3^ *N* ^1^,*N* ^2^:*N* ^2^]bis­[(tetra­hydro­furan)lithium], [Li_2_(C_18_H_37_N_4_)_2_(C_4_H_8_O)_2_], (**2**), and [bis­(tetra­hydro­furan)­lithium]-di-μ-chlorido-{(*N*,*N*′-di­cyclo­hexyl-1-butyl­amidinato-κ^2^ *N* ^1^,*N* ^2^)[*N*,*N*′,*N*′′,*N*′′′-tetra­cyclo­hexyl-1-(1-butyl­amidinato)guanidinato-κ^2^ *N* ^1^,*N* ^2^]holmate(III)}, [HoLiCl_2_(C_4_H_8_O)_2_(C_17_H_31_N_2_)(C_30_H_53_N_4_)], (**3**). The novel lithium amidino­guanidinate precursors Li[^*n*^BuC(=N*R*)(N*R*)C(N*R*)_2_] [**1**: *R* = Cy (cyclo­hex­yl), **2**: *R* = ^*i*^Pr) were obtained by treatment of *N*,*N*′-diorganocarbodi­imides, *R*—N=C=N—*R* (*R* = ^*i*^Pr, Cy), with 0.5 equivalents of *n*-butyl­lithium under well-defined reaction conditions. An X-ray diffraction study of **2** revealed a ladder-type dimeric structure in the solid state. Reaction of anhydrous holmium(III) chloride with *in situ*-prepared **2** afforded the unexpected holmium ‘ate’ complex [^*n*^BuC(=NCy)(NCy)C(NCy)_2_]Ho[^*n*^BuC(NCy)_2_](μ-Cl)_2_Li(THF)_2_ (**3**) in 71% yield. An X-ray crystal structure determination of **3** showed that this complex contains both an amidinate ligand and the new amidino­guanidinate ligand.

## Abstract 2

The first metal complexes comprising amidino­guanidinate ligands have been prepared and structurally characterized, namely bis­[μ-*N*,*N*′,*N*′′,*N*′′′-tetra­isopropyl-1-(1-butyl­amidinato)guanidinato-κ^3^ *N* ^1^,*N* ^2^:*N* ^2^) bis­[(tetra­hydro­furan)­lithium] and [bis­(tetra­hydro­furan)­lithium]-di-μ-chlorido-[*N*,*N*′,*N*′′,*N*′′′-tetra­cyclo­hexyl-1-(1-butyl­amidinato)guanidinato-κ^2^ *N* ^1^,*N* ^2^](*N*,*N*′-di­cyclo­hexyl-1-butyl­amidinato-κ^2^ *N* ^1^,*N* ^2^)holmate(III).

## Chemical context   

**Figure 1:**
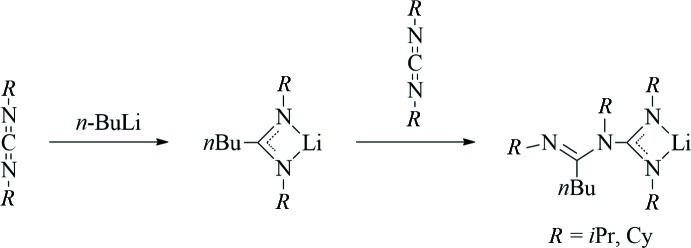
Reaction scheme: Formation of amidino­guanidinate ligands from the reaction of *n*-butyl­lithium with excess carbodi­imide.

Anionic *N*-chelating donor ligands such as the amidinates [*R*C(N*R*)_2_]^−^ and the guanidinates [*R*_2_NC(N*R*)_2_]^−^ have gained tremendous importance in various fields of organometallic and coordination chemistry during the past two decades. Both types of *N*-chelating ligands are often regarded as ‘steric cyclo­penta­dienyl equivalents’ (Bailey & Pace, 2001 ▸; Collins, 2011 ▸; Edelmann, 2013 ▸). Meanwhile, amidinato and guanidin­ato complexes are known for virtually every metallic element in the Periodic Table ranging from lithium to uranium (Edelmann, 2009 ▸, 2012 ▸, 2013 ▸; Trifonov, 2010 ▸). Amidinate and guanidinate ligands have been successfully employed in the stabilization of unusual oxidation states such as magnesium(I) and iron(I) as well as the design of various homogeneous catalysts (Collins, 2011 ▸; Edelmann, 2013 ▸). Alkyl-substituted amidinate and guanidinate complexes of various metals have also been established as ALD and MOCVD precursors for the deposition of thin layers of metals, metal oxides, metal nitrides *etc.* (Devi, 2013 ▸). Formally, the amidinate anion is the nitro­gen analogue of the carboxyl­ate anion, while guanidinates are similarly related to the carbamates. However, in contrast to the carboxyl­ates and carbamates, the steric properties of amidinates and guanidinates can be widely tuned through the use of different substituents, both at the outer nitro­gen atoms as well as at the central carbon atom of the NCN unit. Lithium amidinates are normally prepared in a straightforward manner by addition of lithium alkyls to *N*,*N*′-diorganocarbodi­imides in a 1:1 molar ratio, while lithium guanidinates are formed when lithium-*N,N*-di­alkyl­amides are added to *N*,*N*′-diorgano­carbo­di­imides (Stalke *et al.*, 1992 ▸; Aharonovich *et al.*, 2008 ▸; Chlupatý *et al.*, 2011 ▸; Nevoralová *et al.*, 2013 ▸; Hong *et al.*, 2013 ▸). All these reactions are generally quite straightforward and afford the desired products in high yields. We have now discovered that, under certain conditions, reactions of lithium alkyls with *N*,*N*′-diorganocarbodi­imides can afford different products which can be named ‘amidino­guanidinates’ (*cf.* reaction scheme, Fig. 1 ▸). These can even become the major reaction products when the stoichiometry of the reactands is changed from 1:1 to 1:2, *i.e.* when the *N*,*N*′-diorganocarbodi­imide is used in a twofold molar excess. We report here the synthesis and characterization of the first metal complexes comprising ‘amidino­guanidinate’ ligands which can be viewed as dimers of the amidinate anions. The first amidino­guanidinate complexes described here include the lithium precursors Li[^*n*^BuC(=N*R*)(N*R*)C(N*R*)_2_] (**1**: *R* = Cy (cyclo­hex­yl), **2**: *R* = ^*i*^Pr) and the holmium(III) ‘ate’ complex [^*n*^Bu-C(=NCy)(NCy)C(NCy)_2_]Ho[^*n*^BuC(NCy)_2_](μ-Cl)_2_Li(THF)_2_ (**3**).
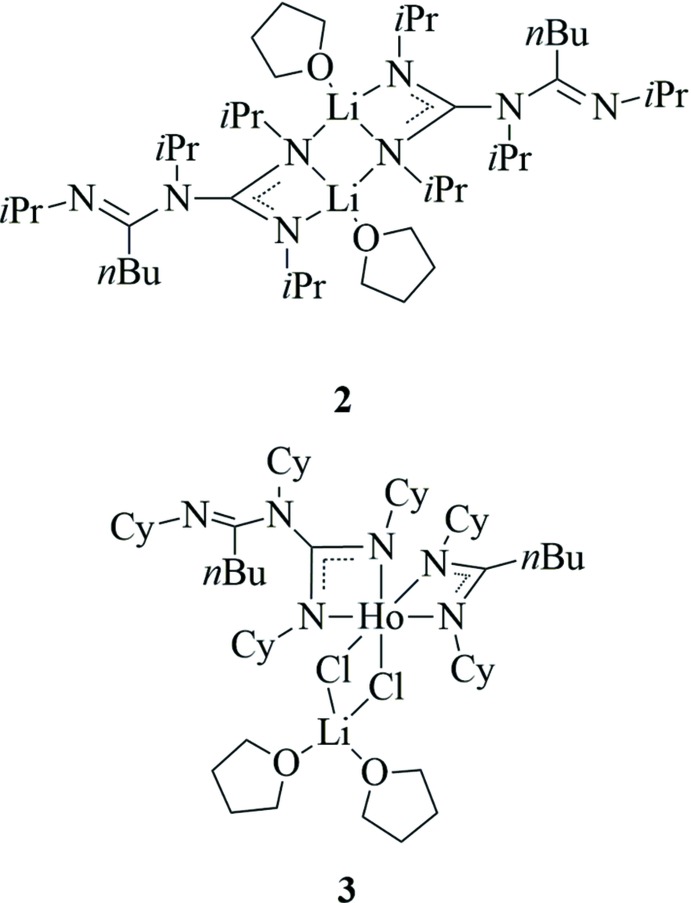


A reaction between *N*,*N*′-di­cyclo­hexyl­carbodi­imide and ^*n*^BuLi in a 2:1 molar ratio in THF afforded the first lithium amidino­guanidinate, Li[^*n*^BuC(=NCy)(NCy)C(NCy)_2_]·THF (**1**), in 60% yield. This reaction represents the first case of dimerization of a carbodi­imide under formation of a novel amidino­guanidinate anion. The lithium-amidino­guanidinate salt **1** is partially soluble in THF, Et_2_O, and DME and slightly soluble even in toluene and *n*-pentane. The new sterically bulky amidino­guanidinate **1** has been fully characterized by spectroscopic methods and elemental analysis to confirm the product as shown in Fig. 1 ▸. DMSO-*d*_6_ (DMSO = dimethyl sulfoxide) was found to be the best solvent for measuring the NMR spectra of Li[^*n*^BuC(=NCy)(NCy)C(NCy)_2_]·THF. A mass spectrum of **1** showed only fragments for the monomeric compound. Inter­estingly, the reaction using *N*,*N*′-di­cyclo­hexyl­carbodi­imide and *n*-butyl­lithium in THF according to Fig. 1 ▸ represents the only case thus far where a pure amidino­guanidinate salt (**1**) could be isolated. A similar reaction carried out with *N*,*N*′-diiso­propyl­carbodi­imide produced the isopropyl-substituted amidino­guanidinate salt **2** in >70% yield, although NMR data indicated the presence of significant amounts of an impurity, presumably the ‘normal’ lithium amidinate Li[^*n*^BuC(N^*i*^Pr)_2_], which could not be separated by fractional crystallization from solvents like THF, DME or diethyl ether. However, occasionally a small amount of well-formed single-crystals of **2** were obtained directly from the reaction mixture which allowed a structural characterization of the new amidino­guanidinates through X-ray diffraction. Apparently the formation of the new amidinoguanidinate anions is critically influenced not only by the stoichiometric ratio of the starting materials, but also by the substituents at the *N*-atoms and the solvents employed. The solvent effect became apparent when reactions of *N*,*N*′-di­cyclo­hexyl­carbodi­imide with 0.5 or 0.3 equiv. of *n*-butyl­lithium were carried out in Et_2_O solution. Using this solvent, the reactions produced a variable mixture of amidino­guanidinate and amidinate salts, Li[^*n*^BuC(=NCy)(NCy)C(NCy)_2_] and Li[^*n*^BuC(NCy)_2_], respectively, as illustrated in the reaction scheme (Fig. 1 ▸). This was clearly indicated by the rather ‘messy’ NMR spectra of the reaction products. Attempts to separate the product mixture by fractional crystallization from THF, DME, or diethyl ether were unsuccessful.

The presence of both types of anions in the reaction mixture obtained was also confirmed by the subsequent reaction of the *in situ*-prepared mixture of Li[^*n*^BuC(=NCy)(NCy)C(NCy)_2_] and Li[^*n*^BuC(NCy)_2_] with anhydrous HoCl_3_. In detail, treatment of *N*,*N*′-di­cyclo­hexyl­carbodi­imide with 0.5 equiv. of ^*n*^BuLi in Et_2_O followed by addition of anhydrous HoCl_3_ (Freeman & Smith, 1958 ▸) in THF produced a yellow solution. Separation of the LiCl by-product and recrystallization from *n*-pentane afforded the unexpected holmium complex [^*n*^BuC(=NCy)(NCy)C(NCy)_2_]Ho[^*n*^BuC(NCy)_2_](*μ*-Cl)_2_Li(THF)_2_ (**3**) in 71% yield. This compound is a mixed-ligand complex containing both the new amidino­guanidinato ligand and the normal amidinato ligand [^*n*^BuC(NCy)_2_]^−^ in the coordination sphere of holmium. Compound **3** was fully characterized by its IR spectrum, elemental analysis and single-crystal X-ray diffraction. As a result of the highly paramagnetic nature of the Ho^3+^ ion, it was impossible to obtain inter­pretable NMR data for **3**. Yellow, air- and moisture-sensitive, needle-like single-crystals of **3** were obtained by slowly cooling a saturated solution in *n*-pentane to 268 K.

In summarizing the results reported here, we prepared the first metal complexes containing novel amidino­guanidinate ligands obtained by dimerization of *N*,*N*′-diorganocarbodi­imides in the presences of sub-stoichiometric amounts of *n*-butyl­lithium. The cyclo­hexyl-substituted lithium-amidino­guanidinate salt Li[^*n*^BuC(=NCy)(NCy)C(NCy)_2_]·THF (**1**) is readily available as a pure solid in fairly good yield (60%). This compound could play an inter­esting role as a precursor for the synthesis of new transition metal and lanthanide amidino­guanidinate complexes. The first lanthanide complex comprising the new ligand system is the holmium ‘ate’ complex [^*n*^BuC(=NCy)(NCy)C(NCy)_2_]Ho[^*n*^BuC(NCy)_2_](*μ*-Cl)_2_Li(THF)_2_ (**3**).

## Structural commentary   

**Figure 2:**
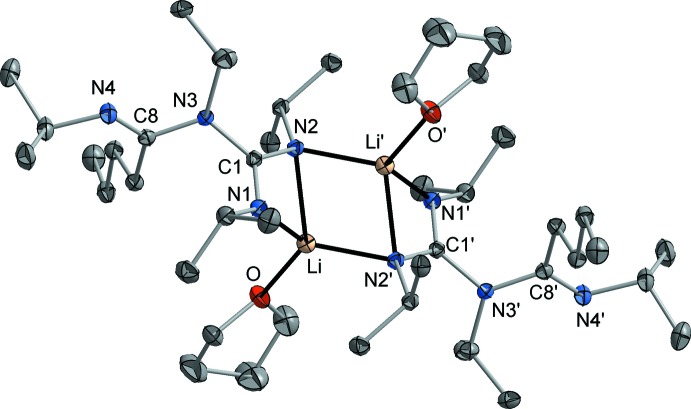
The mol­ecular structure of compound **2** in the crystal. Displacement ellipsoids are drawn at the 50% probability level and H atoms have been omitted for clarity. Symmetry operator to generate equivalent atoms: 2 − *x*, 1 − *y*, −*z*.

The crystal structure determination of **2** revealed the presence of ladder-type centrosymmetric dimers (space group *P*2_1_/c, *Z* = 2), which is the most characteristic structural motif of most previously characterized lithium amidinates and guanidinates (Stalke *et al.*, 1992 ▸; Snaith & Wright, 1995 ▸; Downard & Chivers, 2001 ▸). Fig. 2 ▸ shows the mol­ecular structure of compound **2**, while crystallographic data are summarized in Table 1 ▸. The central building unit of the dimer is a typical planar Li_2_N_2_ ring, formed by μ-bridging coordination of one of the guanidinate N atoms (N2). The Li—N distances within this ring are 2.0528 (17) and 2.1559 (17) Å and therefore in the expected range. The second N atom of the guanidinate unit (N1) is attached to only one Li atom with a shorter Li—N bond of 2.0177 (18) Å. Through this μ-κ^3^ *N*,*N′*:*N*-coordination mode of the guanidinato moiety, a ‘ladder’ consisting of three four-membered rings is formed. By coordination of a solvent THF mol­ecule, a typical distorted tetra­hedral coordination of the Li atom is completed. The free N donor of the amidinate unit (N4) does not contribute to coordinative saturation of the Li atom. The bonds C1—N1 [1.3197 (12) Å] and C1—N2 [1.3396 (11) Å] are similar in length, indicating a common delocalization of the negative charge within the Li-coordin­ating N–C–N fragment. By contrast, the third C—N bond of the guanidinate unit C1—N3 is considerably longer at 1.4528 (11) Å and can therefore be inter­preted as a pure single bond. The 1-butyl­amidinate fragment does not show any delocalization of the π-electron density, with one distinct double bond [C8—N4, 1.2808 (12) Å] and one single bond [C8—N3, 1.3940 (11) Å]. The amidinate C3–C8–N3 fragment is twisted out of the guanidinate C1/N1/N2/N3 plane by approx. 75°, similar to that found earlier for this type of ligands (Zhou *et al.*, 1998 ▸; Wood *et al.*, 1999 ▸; Lu *et al.*, 2001 ▸).

**Figure 3:**
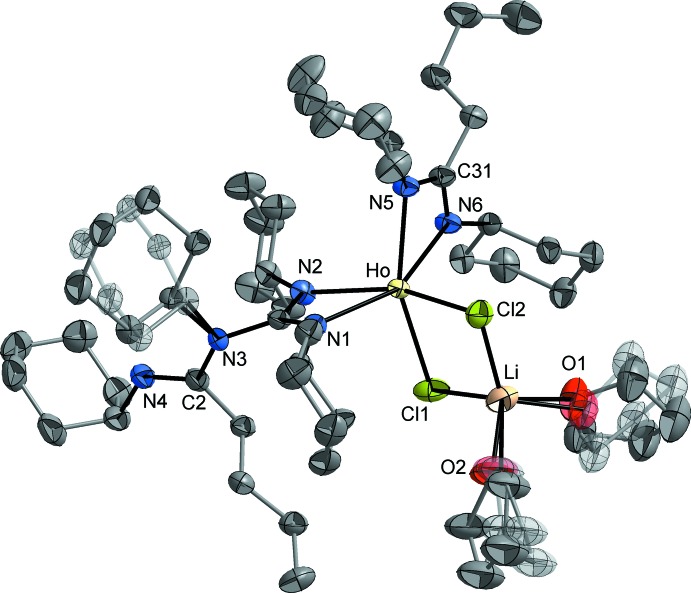
The mol­ecular structure of compound **3** in the crystal, illustrating the disorder of one cyclo­hexyl group and both THF ligands. Displacement ellipsoids drawn at the 50% probability level and H atoms have been omitted for clarity.

The holmium complex **3** crystallizes in the triclinic space group *P* 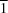 with one mol­ecule in the asymmetric unit. The mol­ecular structure is shown in Fig. 3 ▸. The X-ray diffraction study revealed the presence of an ‘ate’ complex formed through retention of a [LiCl(THF)_2_] fragment by the five-coordinate unit [^*n*^BuC(=NCy)(NCy)C(NCy)_2_]Ho[^*n*^BuC(NCy)_2_]Cl. The phenomenon of ‘ate’ complex formation *via* retention of alkali metal halides in the products is quite common in organolanthanide chemistry (Edelmann, 2006 ▸). It can be traced back to the strong tendency of the large *Ln* ^3+^ ions to adopt high coordination numbers. In the resulting six-coordinate bimetallic complex **3**, the central holmium(III) ion is coordinated by two μ-bridging chloride ions, one chelating amidino­guanidinate ligand and one chelating amidinate ligand. The Ho atom is located in the C1N1N2N3 plane of the amidino­guanidinate ligand and, just like in the case of the lithium derivative **2**, the amidinate N atom N4 does not contribute to metal coordination. The Ho—N distances are in a narrow range of 2.327 (3)–2.354 (3) Å that is in good agreement with the values observed in related lanthanide amidinate and guanidinate complexes (Edelmann, 2009 ▸, 2012 ▸). The same applies to the corresponding coordination angles N1—Ho—N2 [57.0 (1)°] and N5—Ho—N6 [57.3 (1)°]. The guanidinate and the amidinate moiety in compound **3** are arranged nearly perpendicular to each other, gaining a minimal contact between the bulky cyclo­hexyl substituents. The [LiCl_2_(THF)_2_] fragment is attached to the Ho atom in a formally chelating mode, leading to the formation of a regular kite-shaped Ho/Cl1/Li/Cl2 ring [Ho—Cl 2.6326 (13) and 2.6453 (15) Å, Ho—Cl—Li 87.0 (2) and 88.0 (3)°]. The Li atom exhibits a typical tetra­hedral coordination by the two μ-bridging Cl atoms and two THF ligands. Within the chelating NCN units of the amidinato and the amidino­guanidinato ligands, the C—N distances are nearly equal [1.324 (5)–1.336 (5) Å], indicating a typical π-electron delocal­ization within these units. The conformation of the amidinatoguanidinate ligand is very similar to that in compound **2** (angle between guanidinate and amidinate plane approx. 75°), and the localization of single and double bonds within the 1-butyl­amidinate backbone is identical with that in the lithium derivative [C—N 1.272 (5)–1.429 (5) Å].

## Supra­molecular features   

Due to an effective ‘packaging’ of the mol­ecules by the sterically demanding alkyl substituents, both title compounds do not feature any specific inter­molecular inter­actions. In the lithium derivative **2**, the closest inter­molecular contacts are between two isopropyl-CH_3_ groups [C3⋯C10, 3.740 (3) Å] and between an isopropyl-CH_3_ and a butyl-CH_3_ group [C13⋯C18, 3.744 (4) Å]. The crystal structure of the holmium complex **3** comprises a close package of cyclo­hexyl groups, butyl groups and THF ligands with a minimal H_2_C⋯CH_2_ distance of 3.64 (4) Å, and one H_2_C⋯CH_3_ contact of at least 3.73 (6) Å (C6 and C21*B* of disordered cyclo­hexyl group).

## Database survey   

For other structurally characterized lithium amidinates and guanidinates, see: Stalke *et al.* (1992 ▸), Aharonovich *et al.* (2008 ▸), Chlupatý *et al.* (2011 ▸), Nevoralová *et al.* (2013 ▸) and Hong *et al.* (2013 ▸).

For other lanthanide(III) complexes with amidinate ligands, see: Richter *et al.* (2004 ▸), Edelmann (2009 ▸, 2012 ▸) and Deacon *et al.* (2014 ▸).

## Synthesis and crystallization   

**General Procedures:** All reactions were carried out under an inert atmosphere of dry argon employing standard Schlenk and glovebox techniques. THF and *n*-pentane were distilled from sodium/benzo­phenone under nitro­gen atmosphere prior to use. All glassware was oven-dried at 393 K for at least 24 h, assembled while hot, and cooled under high vacuum prior to use. Anhydrous holmium(III) chloride was prepared according to the literature method (Freeman & Smith, 1958 ▸). *n*-Butyl­lithium solution, *N*,*N*′-diiso­propyl­carbodi­imide and *N*,*N*′-di­cyclo­hexyl­carbodi­imide were purchased from Aldrich and used as received. ^1^H NMR (400 MHz) and ^13^C NMR (100.6 MHz) spectra were recorded in DMSO-*d_6_* solution on a Bruker DPX 400 spectrometer at 298 K. Chemical shifts are referenced to TMS. IR spectra were recorded using KBr pellets on a Perkin Elmer FT–IR spectrometer system 2000 between 4000 cm^−1^ and 400 cm^−1^. Microanalyses (C, H and N) of compounds **1** and **3** were performed using a Leco CHNS 932 apparatus.

**Synthesis of Li[** ***^n^*** **BuC(=NCy)(NCy)C(NCy)_2_]·THF (1):** A solution of *N*,*N*′-di­cyclo­hexyl­carbodi­imide (10.30 g, 50 mmol) in 100 ml of THF at 253 K was treated slowly with *n*-butyl­lithium (16 ml, 1.6 *M* solution in hexa­nes). The reaction mixture was stirred for 10 min at 253 K, then warmed to room temperature and stirred overnight to give a white suspension in THF. The solvent was removed under vacuum affording **1** as white solid. Yield: 16.4 g, 60%. Elemental analysis for C_34_H_61_LiN_4_O (548.83 g mol^−1^): C, 74.41; H, 11.20; N, 10.21; found C, 74.82; H, 10.85; N, 10.50. ^1^H NMR (400 MHz, (CD_3_)_2_SO, 298 K): *δ* (p.p.m.) 3.84 (*m*, 1H, CH, Cy), 3.60 (*m*, 4H, THF), 3.43 (*m*, 1H, CH, Cy), 3.04–3.18 (*m*, 2H, CH, Cy), 2.66 (*m*, 1H, CH_2_, ^*n*^Bu), 2.33 (*m*, 1H, CH_2_, ^*n*^Bu), 2.09 (*m*, 2H, CH_2_, ^*n*^Bu), 1.84 (*m*, 2H, CH_2_, ^*n*^Bu), 1.76 (*m*, 4H, THF), 1.65 (*m*, 8H, CH_2_, Cy), 1.52 (*m*, 6H, CH_2_, Cy), 1.26 (*m*, 26H, CH_2_, Cy), 0.85 (*m*, 3H, CH_3_, ^*n*^Bu); ^13^C NMR (100.6 MHz, C_6_D_6_, 298 K): *δ* (p.p.m.) 155.3 (NCN), 145.1 (NCN), 67.0 (THF), 55.4 (CH, Cy), 54.2 (CH, Cy), 49.3 (CH, Cy), 35.7 (CH_2_, Cy), 35.1 (CH_2_, Cy), 34.8 (CH_2_, Cy), 34.5 (CH_2_, ^*n*^Bu), 30.7 (CH_2_, ^*n*^Bu), 29.5 (CH_2_, ^*n*^Bu), 25.8 (THF), 24.9 (CH_2_, Cy), 22.6 (CH_2_, Cy), 22.1 (CH_2_, Cy), 13.8 (CH_3_). MS (EI, *M* = 548.50): *m*/*z* (%) 125.2 (27) [Cy + C_3_H_6_]^+^, 153.2 (88) [2Cy − Me]^2+^, 183.3 (20) [2Cy + Me]^2+^, 207.3 (12) [C(NCy)_2_]^+^, 222.3 (62) [C(NCy)_2_ + Me]^2+^, 235.4 (100) [C(NCy)_2_ + C_2_H_5_]^+^, 264.4 (55) [^*n*^Bu + C(NCy)_2_]^+^. IR (KBr): *n* (cm^−1^) 3449 (*w*), 3327 (*w*), 3225 (*w*), 2927 (*vs*), 2853 (*s*), 2666 (*w*), 2533 (*w*), 2354 (*w*), 2120 (*w*), 1959 (*w*), 1645 (*m*), 1578 (*w*), 1516 (*m*), 14450 (*m*), 1367 (*w*), 1339 (*m*), 1155 (*w*), 1128 (*m*), 1105 (*w*), 1053 (*w*), 1029 (*w*), 988 (*w*), 919 (*w*), 889 (*w*), 845 (*w*), 804 (*w*), 748 (*w*), 695 (*w*), 657 (*w*), 640 (*w*), 555 (*w*), 502 (*w*), 454 (*w*).

**Synthesis of Li[** ***^n^*** **BuC(=N** ***^i^*** **Pr)(N** ***^i^*** **Pr)C(N** ***^i^*** **Pr)_2_]·THF (2):** In a similar manner as for compound **1**, *N*,*N*′-diiso­propyl­carbodi­imide (4.2 g, 50 mmol) was treated with *n*-butyl­lithium (10 ml, 2.5 *M* solution in hexa­nes) in THF solution (80 ml). From this reaction 14.3 g of colorless **2** were isolated. X-ray quality single crystals (colorless rods) were occasionally obtained directly upon cooling of the reaction mixture to 278 K. However, NMR data showed that the bulk product was heavily contaminated with the lithium amidinate salt Li[^*n*^BuC(N^*i*^Pr)_2_] (10–20%) which could not be separated by fractional crystallization.

**Synthesis of [** ***^n^*** **BuC(=NCy)(NCy)C(NCy)_2_]Ho[** ***^n^*** **BuC(NCy)_2_](**μ**-Cl)_2_Li(THF)_2_ (3):** A solution of anhydrous HoCl_3_ (1.0 g, 3.6 mmol) in 50 ml THF was added to a stirred Et_2_O solution (80 ml) of an *in situ*-prepared mixture of Li[^*n*^Bu-C(=NCy)(NCy)C(NCy)_2_] and Li[^*n*^BuC(NCy)_2_] (*N*,*N*′-di­cyclo­hexyl­carbodi­imide (10.30 g, 50 mmol) in 80 ml of Et_2_O and was treated slowly with *n*-butyl­lithium (16 mL, 1.6 *M* solution in hexa­nes) at 253 K. The reaction mixture was stirred for 3 h at room temperature. The solvents were evaporated under vacuum, and the residue was extracted with 20 ml *n*-pentane. Concentration and cooling of the filtered solution to 278 K afforded **3** as yellow, air- and moisture-sensitive, needle-like crystals. Yield: 2.8 g, 71%. Elemental analysis for C_55_H_100_Cl_2_HoLiN_6_O_2_ (1120.22 g mol^−1^): C, 58.97; H, 9.00; N, 7.50; found C, 58.92; H, 8.98; N, 7.44%. IR (KBr): *n* (cm^−1^) 3321 (*w*), 3223 (*w*), 2929 (*vs*), 2857 (*s*), 2661 (*w*), 2525 (*w*), 2356 (*w*), 2118 (*w*), 1952 (*w*), 1577 (*w*), 1518 (*m*), 1367 (*w*), 1156 (*w*), 1129 (*m*), 1108 (*w*), 1085 (*w*), 1055 (*w*), 1045 (*w*), 983 (*w*), 922 (*w*), 892 (*w*), 865 (*w*), 820 (*w*), 715 (*w*), 657 (*w*), 643 (*w*), 553 (*w*), 505 (*w*), 456 (*w*). Meaningful NMR spectra could not be obtained due to the strong paramagnetism of the Ho^3+^ ion.

## Refinement   

Crystal data, data collection and structure refinement details are summarized in Table 1 ▸. All H atoms were fixed geometrically and refined using a riding model with *U*_iso_(H) = 1.2 *U*_eq_(C). C—H distances in CH_3_ groups were constrained to 0.98 Å, those in CH_2_ groups to 0.99 Å and those in CH groups to 1.00 Å. Methyl H atoms were allowed to rotate around the C—C vector but not to tip to best fit the experimental electron density (AFIX 137 in *SHELXL*). In the crystallographic dataset of compound **3**, the intensities of reflections (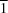
11) and (1
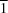
1) strongly disagreed with the structural model and were therefore omitted from the refinement. One of the cyclo­hexyl groups (C19–C24) and both THF ligands (O1, C48–C51 and O2, C52–C55) in compound **3** are disordered. The aforementioned atoms were each split over two sites (site occupancy factors refined freely). Equivalent disordered THF and cyclo­hexyl moieties were restrained to have similar geometries (SAME restraint in *SHELXLL*), and *U*_ij_ components of ADPs were restrained to be similar for atoms closer than 1.7 Å (SIMU restraint in *SHELXL*; the esd applied was 0.01 Å^2^). Occupancy ratios refined to 0.760 (6) and 0.240 (6) for the cyclo­hexyl group (C19–C24), and to 0.663 (11) and 0.337 (11) (O1, C48–C51) and to 0.823 (11) and 0.177 (11) (O2, C52–C55) for the THF moieties.

## Supplementary Material

Crystal structure: contains datablock(s) compound_2, compound_3. DOI: 10.1107/S2056989016015322/zl2679sup1.cif

Structure factors: contains datablock(s) compound_2. DOI: 10.1107/S2056989016015322/zl2679compound_2sup2.hkl

Structure factors: contains datablock(s) compound_3. DOI: 10.1107/S2056989016015322/zl2679compound_3sup3.hkl

CCDC references: 1414136, 1414137

Additional supporting information:  crystallographic information; 3D view; checkCIF report

## Figures and Tables

**Table 1:** Experimental details

	**2**	**3**
Crystal data		
Chemical formula	[Li_2_C_18_H_37_N_4_)_2_(C_4_H_8_O)_2_]	[LiHoCl_2_(C_4_H_8_O)_2_(C_17_H_31_N_2_)(C_30_H_53_N_4_)]
*M*_r_	777.11	1120.17
Crystal system, space group	Monoclinic, *P*2_1_/*c*	Triclinic, *P* 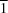
Temperature (K)	100	153
*a*, *b*, *c* (Å)	9.93297 (7), 13.7239 (1), 18.07940 (13)	12.909 (3), 15.095 (3), 16.786 (3)
α, β, γ (°)	90, 92.8380 (6), 90	100.67 (3), 97.20 (3), 109.50 (3)
*V* (Å^3^)	2461.54 (3)	2967.5 (12)
*Z*	2	2
Radiation type	Cu *K*α	Mo *K*α
μ (mm^−1^)	0.49	1.47
Crystal size (mm)	0.18 × 0.12 × 0.04	0.34 × 0.20 × 0.12

Data collection		
Diffractometer	Agilent Xcalibur, Atlas, Nova	Stoe *IPDS* 2T
Absorption correction	Multi-scan (*CrysAlis PRO*; Agilent, 2011 ▸)	For a sphere (*X-AREA* and *X-RED*; Stoe & Cie, 2002 ▸)
*T*_min_, *T*_max_	0.891, 1.000	0.814, 0.889
No. of measured, independent and observed [*I* > 2σ(*I*)] reflections	30246, 5126, 4657	29449, 12948, 9625
*R*_int_	0.027	0.078
(sin θ/λ)_max_ (Å^−1^)	0.629	0.639

Refinement		
*R*[*F* ^2^ > 2σ(*F* ^2^)], *wR*(*F* ^2^), *S*	0.036, 0.096, 1.05	0.048, 0.074, 0.91
No. of reflections	5126	12948
No. of parameters	263	751
No. of restraints	0	552
H-atom treatment	H-atom parameters constrained	H-atom parameters constrained
Δρ_max_, Δρ_min_ (e Å^−3^)	0.31, −0.21	0.98, −1.68

Computer programs: *CrysAlis PRO* (Agilent, 2011 ▸), *X-AREA* and *X-RED* (Stoe & Cie, 2002 ▸), *SHELXS97* (Sheldrick, 2008 ▸), *SHELXL2016* (Sheldrick, 2015 ▸), *DIAMOND* (Brandenburg, 1999 ▸) and *publCIF* (Westrip, 2010 ▸).

## supplementary crystallographic information

### (compound_2) Bis[µ-N,N',N'',N'''-tetraisopropyl-1-(1-butylamidinato)guanidinato-κ3N1,N2:N2]bis[(tetrahydrofuran)lithium] Crystal data

**Table:**

[Li_2_C_18_H_37_N_4_)_2_(C_4_H_8_O)_2_]	*F*(000) = 864
*M**_r_* = 777.11	*D*_x_ = 1.048 Mg m^−^^3^
Monoclinic, *P*2_1_/*c*	Cu *K*α radiation, λ = 1.54184 Å
*a* = 9.93297 (7) Å	Cell parameters from 18888 reflections
*b* = 13.7239 (1) Å	θ = 3.2–75.6°
*c* = 18.07940 (13) Å	µ = 0.49 mm^−^^1^
β = 92.8380 (6)°	*T* = 100 K
*V* = 2461.54 (3) Å^3^	Plate, colorless
*Z* = 2	0.18 × 0.12 × 0.04 mm

### (compound_2) Bis[µ-N,N',N'',N'''-tetraisopropyl-1-(1-butylamidinato)guanidinato-κ3N1,N2:N2]bis[(tetrahydrofuran)lithium] Data collection

**Table:**

Agilent Xcalibur, Atlas, Nova diffractometer	5126 independent reflections
Radiation source: Nova (Cu) X-ray Source	4657 reflections with *I* > 2σ(*I*)
Detector resolution: 10.3543 pixels mm^-1^	*R*_int_ = 0.027
ω scans	θ_max_ = 75.8°, θ_min_ = 4.1°
Absorption correction: multi-scan (CrysAlisPro; Agilent, 2011)	*h* = −12→11
*T*_min_ = 0.891, *T*_max_ = 1.000	*k* = −16→17
30246 measured reflections	*l* = −17→22

### (compound_2) Bis[µ-N,N',N'',N'''-tetraisopropyl-1-(1-butylamidinato)guanidinato-κ3N1,N2:N2]bis[(tetrahydrofuran)lithium] Refinement

**Table:**

Refinement on *F*^2^	Secondary atom site location: difference Fourier map
Least-squares matrix: full	Hydrogen site location: inferred from neighbouring sites
*R*[*F*^2^ > 2σ(*F*^2^)] = 0.036	H-atom parameters constrained
*wR*(*F*^2^) = 0.096	*w* = 1/[σ^2^(*F*_o_^2^) + (0.0474*P*)^2^ + 0.6355*P*] where *P* = (*F*_o_^2^ + 2*F*_c_^2^)/3
*S* = 1.05	(Δ/σ)_max_ = 0.001
5126 reflections	Δρ_max_ = 0.31 e Å^−^^3^
263 parameters	Δρ_min_ = −0.21 e Å^−^^3^
0 restraints	Extinction correction: SHELXL2016 (Sheldrick, 2015), Fc^*^=kFc[1+0.001xFc^2^λ^3^/sin(2θ)]^-1/4^
Primary atom site location: structure-invariant direct methods	Extinction coefficient: 0.00067 (16)

### (compound_2) Bis[µ-N,N',N'',N'''-tetraisopropyl-1-(1-butylamidinato)guanidinato-κ3N1,N2:N2]bis[(tetrahydrofuran)lithium] Special details

**Table:**

Geometry. All esds (except the esd in the dihedral angle between two l.s. planes) are estimated using the full covariance matrix. The cell esds are taken into account individually in the estimation of esds in distances, angles and torsion angles; correlations between esds in cell parameters are only used when they are defined by crystal symmetry. An approximate (isotropic) treatment of cell esds is used for estimating esds involving l.s. planes.

### (compound_2) Bis[µ-N,N',N'',N'''-tetraisopropyl-1-(1-butylamidinato)guanidinato-κ3N1,N2:N2]bis[(tetrahydrofuran)lithium] Fractional atomic coordinates and isotropic or equivalent isotropic displacement parameters (Å^2^)

**Table:**

	*x*	*y*	*z*	*U*_iso_*/*U*_eq_	
C1	1.13747 (9)	0.49827 (6)	0.11713 (5)	0.01508 (18)	
C2	0.98312 (9)	0.49701 (7)	0.21715 (5)	0.0199 (2)	
H2	1.067737	0.513701	0.246717	0.024*	
C3	0.92362 (11)	0.40357 (8)	0.24810 (6)	0.0286 (2)	
H3A	0.896871	0.415184	0.298801	0.034*	
H3B	0.991346	0.351631	0.248263	0.034*	
H3C	0.844533	0.384025	0.217059	0.034*	
C4	0.88214 (10)	0.58050 (8)	0.22330 (6)	0.0269 (2)	
H4A	0.866222	0.592480	0.275587	0.032*	
H4B	0.796995	0.562914	0.196968	0.032*	
H4C	0.918553	0.639559	0.201291	0.032*	
C5	1.28751 (9)	0.48998 (7)	0.01575 (5)	0.01694 (19)	
H5	1.355755	0.487672	0.058274	0.020*	
C6	1.28738 (10)	0.39198 (7)	−0.02470 (5)	0.0216 (2)	
H6A	1.376825	0.379957	−0.043384	0.026*	
H6B	1.220220	0.393392	−0.066259	0.026*	
H6C	1.265025	0.339863	0.009657	0.026*	
C7	1.32471 (9)	0.57220 (7)	−0.03661 (5)	0.0223 (2)	
H7A	1.415937	0.561386	−0.053232	0.027*	
H7B	1.321415	0.634711	−0.010560	0.027*	
H7C	1.260605	0.573175	−0.079593	0.027*	
C8	1.27924 (9)	0.42776 (7)	0.21963 (5)	0.01661 (18)	
C9	1.32067 (9)	0.59902 (7)	0.17680 (5)	0.01914 (19)	
H9	1.308813	0.631572	0.127416	0.023*	
C10	1.25452 (10)	0.66563 (7)	0.23194 (6)	0.0250 (2)	
H10A	1.301263	0.728531	0.233932	0.030*	
H10B	1.260085	0.635471	0.281130	0.030*	
H10C	1.159716	0.675682	0.216188	0.030*	
C11	1.47245 (9)	0.59045 (7)	0.19329 (5)	0.0230 (2)	
H11A	1.515323	0.653447	0.184560	0.028*	
H11B	1.509569	0.541135	0.160805	0.028*	
H11C	1.489873	0.571267	0.245094	0.028*	
C12	1.37114 (11)	0.37049 (7)	0.33586 (5)	0.0240 (2)	
H12	1.353554	0.305706	0.311966	0.029*	
C13	1.28767 (12)	0.38011 (9)	0.40405 (6)	0.0340 (3)	
H13A	1.312206	0.327951	0.439204	0.041*	
H13B	1.191678	0.375085	0.389225	0.041*	
H13C	1.305491	0.443472	0.427535	0.041*	
C14	1.52124 (11)	0.38091 (9)	0.35630 (6)	0.0310 (2)	
H14A	1.549829	0.328604	0.390479	0.037*	
H14B	1.538082	0.444157	0.380172	0.037*	
H14C	1.572284	0.376680	0.311393	0.037*	
C15	1.25689 (9)	0.32623 (7)	0.18708 (5)	0.01830 (19)	
H15A	1.186367	0.329059	0.146475	0.022*	
H15B	1.225324	0.281858	0.225761	0.022*	
C16	1.38830 (9)	0.28650 (7)	0.15713 (5)	0.01958 (19)	
H16A	1.424067	0.334657	0.122370	0.024*	
H16B	1.455792	0.278751	0.198869	0.024*	
C17	1.37014 (10)	0.18882 (7)	0.11733 (5)	0.0215 (2)	
H17A	1.346490	0.138322	0.153569	0.026*	
H17B	1.294556	0.193946	0.079734	0.026*	
C18	1.49719 (11)	0.15769 (8)	0.07957 (6)	0.0281 (2)	
H18A	1.479126	0.097653	0.051461	0.034*	
H18B	1.569961	0.146142	0.117146	0.034*	
H18C	1.524114	0.209274	0.045858	0.034*	
C19	0.94341 (14)	0.23713 (9)	−0.03529 (6)	0.0357 (3)	
H19A	1.029322	0.232716	−0.060603	0.043*	
H19B	0.876981	0.272617	−0.067824	0.043*	
C20	0.89174 (18)	0.13655 (11)	−0.01791 (8)	0.0536 (4)	
H20A	0.932479	0.086723	−0.049483	0.064*	
H20B	0.792403	0.133407	−0.025275	0.064*	
C21	0.93514 (17)	0.12190 (9)	0.06268 (8)	0.0476 (3)	
H21A	0.874625	0.075971	0.087114	0.057*	
H21B	1.028931	0.097574	0.068212	0.057*	
C22	0.92342 (12)	0.22318 (8)	0.09337 (6)	0.0292 (2)	
H22A	0.829573	0.236969	0.106203	0.035*	
H22B	0.983554	0.231476	0.138250	0.035*	
LI	0.96597 (16)	0.42919 (12)	0.03809 (9)	0.0200 (3)	
N1	1.01480 (8)	0.48338 (6)	0.13965 (4)	0.01756 (17)	
N2	1.15401 (7)	0.50792 (5)	0.04440 (4)	0.01578 (16)	
N3	1.25379 (7)	0.50292 (5)	0.16898 (4)	0.01575 (16)	
N4	1.32979 (8)	0.44822 (6)	0.28432 (4)	0.01977 (17)	
O	0.96357 (7)	0.28667 (5)	0.03486 (4)	0.02535 (17)	

### (compound_2) Bis[µ-N,N',N'',N'''-tetraisopropyl-1-(1-butylamidinato)guanidinato-κ3N1,N2:N2]bis[(tetrahydrofuran)lithium] Atomic displacement parameters (Å^2^)

**Table:**

	*U*^11^	*U*^22^	*U*^33^	*U*^12^	*U*^13^	*U*^23^
C1	0.0155 (4)	0.0136 (4)	0.0160 (4)	0.0008 (3)	−0.0011 (3)	−0.0001 (3)
C2	0.0186 (4)	0.0254 (5)	0.0159 (4)	−0.0003 (4)	0.0025 (3)	−0.0002 (3)
C3	0.0315 (5)	0.0328 (6)	0.0221 (5)	−0.0046 (4)	0.0078 (4)	0.0036 (4)
C4	0.0244 (5)	0.0347 (6)	0.0219 (5)	0.0067 (4)	0.0039 (4)	−0.0025 (4)
C5	0.0141 (4)	0.0205 (4)	0.0162 (4)	0.0009 (3)	0.0009 (3)	0.0013 (3)
C6	0.0232 (5)	0.0234 (5)	0.0184 (4)	0.0025 (4)	0.0028 (3)	−0.0005 (4)
C7	0.0182 (4)	0.0249 (5)	0.0241 (5)	−0.0003 (4)	0.0052 (3)	0.0041 (4)
C8	0.0154 (4)	0.0174 (4)	0.0171 (4)	0.0004 (3)	0.0013 (3)	0.0010 (3)
C9	0.0202 (4)	0.0168 (4)	0.0202 (4)	−0.0028 (3)	−0.0013 (3)	0.0001 (3)
C10	0.0253 (5)	0.0203 (5)	0.0293 (5)	0.0001 (4)	0.0005 (4)	−0.0046 (4)
C11	0.0198 (4)	0.0256 (5)	0.0235 (5)	−0.0054 (4)	0.0005 (4)	−0.0033 (4)
C12	0.0322 (5)	0.0203 (5)	0.0189 (5)	0.0034 (4)	−0.0057 (4)	0.0021 (4)
C13	0.0382 (6)	0.0385 (6)	0.0252 (5)	0.0038 (5)	0.0018 (4)	0.0124 (5)
C14	0.0314 (6)	0.0336 (6)	0.0272 (5)	0.0099 (4)	−0.0063 (4)	−0.0002 (4)
C15	0.0193 (4)	0.0166 (4)	0.0188 (4)	−0.0008 (3)	−0.0011 (3)	0.0009 (3)
C16	0.0205 (4)	0.0185 (4)	0.0195 (4)	0.0008 (3)	−0.0012 (3)	−0.0001 (3)
C17	0.0265 (5)	0.0183 (4)	0.0196 (4)	0.0012 (4)	0.0000 (4)	0.0004 (3)
C18	0.0325 (5)	0.0280 (5)	0.0239 (5)	0.0055 (4)	0.0025 (4)	−0.0028 (4)
C19	0.0500 (7)	0.0284 (6)	0.0290 (6)	0.0071 (5)	0.0045 (5)	−0.0049 (4)
C20	0.0773 (11)	0.0365 (7)	0.0462 (8)	−0.0150 (7)	−0.0042 (7)	−0.0097 (6)
C21	0.0688 (9)	0.0217 (6)	0.0518 (8)	−0.0069 (6)	−0.0025 (7)	0.0040 (5)
C22	0.0346 (6)	0.0232 (5)	0.0294 (5)	−0.0059 (4)	−0.0020 (4)	0.0076 (4)
LI	0.0200 (7)	0.0195 (8)	0.0203 (8)	−0.0011 (6)	−0.0004 (6)	0.0019 (6)
N1	0.0161 (4)	0.0217 (4)	0.0150 (4)	−0.0003 (3)	0.0017 (3)	0.0006 (3)
N2	0.0134 (3)	0.0188 (4)	0.0152 (4)	0.0003 (3)	0.0010 (3)	0.0008 (3)
N3	0.0163 (4)	0.0153 (4)	0.0154 (4)	−0.0014 (3)	−0.0019 (3)	0.0008 (3)
N4	0.0234 (4)	0.0190 (4)	0.0166 (4)	0.0018 (3)	−0.0016 (3)	0.0013 (3)
O	0.0318 (4)	0.0186 (3)	0.0258 (4)	−0.0010 (3)	0.0031 (3)	0.0021 (3)

### (compound_2) Bis[µ-N,N',N'',N'''-tetraisopropyl-1-(1-butylamidinato)guanidinato-κ3N1,N2:N2]bis[(tetrahydrofuran)lithium] Geometric parameters (Å, º)

**Table:**

C1—N1	1.3197 (12)	C12—C13	1.5251 (15)
C1—N2	1.3396 (11)	C12—H12	1.0000
C1—N3	1.4528 (11)	C13—H13A	0.9800
C1—LI	2.3667 (18)	C13—H13B	0.9800
C2—N1	1.4633 (11)	C13—H13C	0.9800
C2—C3	1.5296 (14)	C14—H14A	0.9800
C2—C4	1.5304 (13)	C14—H14B	0.9800
C2—H2	1.0000	C14—H14C	0.9800
C3—H3A	0.9800	C15—C16	1.5376 (13)
C3—H3B	0.9800	C15—H15A	0.9900
C3—H3C	0.9800	C15—H15B	0.9900
C4—H4A	0.9800	C16—C17	1.5279 (13)
C4—H4B	0.9800	C16—H16A	0.9900
C4—H4C	0.9800	C16—H16B	0.9900
C5—N2	1.4681 (11)	C17—C18	1.5252 (14)
C5—C7	1.5299 (13)	C17—H17A	0.9900
C5—C6	1.5309 (13)	C17—H17B	0.9900
C5—H5	1.0000	C18—H18A	0.9800
C6—H6A	0.9800	C18—H18B	0.9800
C6—H6B	0.9800	C18—H18C	0.9800
C6—H6C	0.9800	C19—O	1.4441 (13)
C7—H7A	0.9800	C19—C20	1.5111 (18)
C7—H7B	0.9800	C19—H19A	0.9900
C7—H7C	0.9800	C19—H19B	0.9900
C8—N4	1.2808 (12)	C20—C21	1.512 (2)
C8—N3	1.3940 (11)	C20—H20A	0.9900
C8—C15	1.5245 (12)	C20—H20B	0.9900
C9—N3	1.4804 (11)	C21—C22	1.5033 (17)
C9—C10	1.5251 (13)	C21—H21A	0.9900
C9—C11	1.5268 (13)	C21—H21B	0.9900
C9—H9	1.0000	C22—O	1.4419 (12)
C10—H10A	0.9800	C22—H22A	0.9900
C10—H10B	0.9800	C22—H22B	0.9900
C10—H10C	0.9800	LI—O	1.9569 (18)
C11—H11A	0.9800	LI—N1	2.0177 (18)
C11—H11B	0.9800	LI—N2^i^	2.0528 (17)
C11—H11C	0.9800	LI—N2	2.1559 (17)
C12—N4	1.4619 (12)	LI—LI^i^	2.495 (3)
C12—C14	1.5250 (15)	N2—LI^i^	2.0528 (17)
			
N1—C1—N2	118.56 (8)	H14B—C14—H14C	109.5
N1—C1—N3	121.64 (8)	C8—C15—C16	110.49 (7)
N2—C1—N3	119.79 (8)	C8—C15—H15A	109.6
N1—C1—LI	58.42 (6)	C16—C15—H15A	109.6
N2—C1—LI	64.32 (6)	C8—C15—H15B	109.6
N3—C1—LI	158.88 (7)	C16—C15—H15B	109.6
N1—C2—C3	110.39 (8)	H15A—C15—H15B	108.1
N1—C2—C4	109.76 (8)	C17—C16—C15	113.24 (8)
C3—C2—C4	109.55 (8)	C17—C16—H16A	108.9
N1—C2—H2	109.0	C15—C16—H16A	108.9
C3—C2—H2	109.0	C17—C16—H16B	108.9
C4—C2—H2	109.0	C15—C16—H16B	108.9
C2—C3—H3A	109.5	H16A—C16—H16B	107.7
C2—C3—H3B	109.5	C18—C17—C16	112.06 (8)
H3A—C3—H3B	109.5	C18—C17—H17A	109.2
C2—C3—H3C	109.5	C16—C17—H17A	109.2
H3A—C3—H3C	109.5	C18—C17—H17B	109.2
H3B—C3—H3C	109.5	C16—C17—H17B	109.2
C2—C4—H4A	109.5	H17A—C17—H17B	107.9
C2—C4—H4B	109.5	C17—C18—H18A	109.5
H4A—C4—H4B	109.5	C17—C18—H18B	109.5
C2—C4—H4C	109.5	H18A—C18—H18B	109.5
H4A—C4—H4C	109.5	C17—C18—H18C	109.5
H4B—C4—H4C	109.5	H18A—C18—H18C	109.5
N2—C5—C7	110.17 (7)	H18B—C18—H18C	109.5
N2—C5—C6	109.66 (7)	O—C19—C20	106.34 (10)
C7—C5—C6	110.30 (8)	O—C19—H19A	110.5
N2—C5—H5	108.9	C20—C19—H19A	110.5
C7—C5—H5	108.9	O—C19—H19B	110.5
C6—C5—H5	108.9	C20—C19—H19B	110.5
C5—C6—H6A	109.5	H19A—C19—H19B	108.7
C5—C6—H6B	109.5	C19—C20—C21	103.78 (11)
H6A—C6—H6B	109.5	C19—C20—H20A	111.0
C5—C6—H6C	109.5	C21—C20—H20A	111.0
H6A—C6—H6C	109.5	C19—C20—H20B	111.0
H6B—C6—H6C	109.5	C21—C20—H20B	111.0
C5—C7—H7A	109.5	H20A—C20—H20B	109.0
C5—C7—H7B	109.5	C22—C21—C20	102.07 (11)
H7A—C7—H7B	109.5	C22—C21—H21A	111.4
C5—C7—H7C	109.5	C20—C21—H21A	111.4
H7A—C7—H7C	109.5	C22—C21—H21B	111.4
H7B—C7—H7C	109.5	C20—C21—H21B	111.4
N4—C8—N3	119.19 (8)	H21A—C21—H21B	109.2
N4—C8—C15	126.58 (8)	O—C22—C21	104.99 (9)
N3—C8—C15	113.86 (8)	O—C22—H22A	110.7
N3—C9—C10	112.98 (8)	C21—C22—H22A	110.7
N3—C9—C11	112.59 (8)	O—C22—H22B	110.7
C10—C9—C11	111.82 (8)	C21—C22—H22B	110.7
N3—C9—H9	106.3	H22A—C22—H22B	108.8
C10—C9—H9	106.3	O—LI—N1	113.43 (8)
C11—C9—H9	106.3	O—LI—N2^i^	113.13 (8)
C9—C10—H10A	109.5	N1—LI—N2^i^	127.65 (9)
C9—C10—H10B	109.5	O—LI—N2	120.78 (8)
H10A—C10—H10B	109.5	N1—LI—N2	66.33 (6)
C9—C10—H10C	109.5	N2^i^—LI—N2	107.34 (7)
H10A—C10—H10C	109.5	O—LI—C1	115.20 (8)
H10B—C10—H10C	109.5	N1—LI—C1	33.86 (4)
C9—C11—H11A	109.5	N2^i^—LI—C1	130.16 (8)
C9—C11—H11B	109.5	N2—LI—C1	34.06 (4)
H11A—C11—H11B	109.5	O—LI—LI^i^	139.94 (12)
C9—C11—H11C	109.5	N1—LI—LI^i^	98.96 (9)
H11A—C11—H11C	109.5	N2^i^—LI—LI^i^	55.58 (6)
H11B—C11—H11C	109.5	N2—LI—LI^i^	51.76 (6)
N4—C12—C14	109.05 (8)	C1—LI—LI^i^	79.32 (8)
N4—C12—C13	107.78 (8)	C1—N1—C2	121.42 (8)
C14—C12—C13	111.10 (9)	C1—N1—LI	87.72 (7)
N4—C12—H12	109.6	C2—N1—LI	149.90 (8)
C14—C12—H12	109.6	C1—N2—C5	119.11 (7)
C13—C12—H12	109.6	C1—N2—LI^i^	131.28 (8)
C12—C13—H13A	109.5	C5—N2—LI^i^	108.43 (7)
C12—C13—H13B	109.5	C1—N2—LI	81.63 (7)
H13A—C13—H13B	109.5	C5—N2—LI	133.84 (7)
C12—C13—H13C	109.5	LI^i^—N2—LI	72.66 (7)
H13A—C13—H13C	109.5	C8—N3—C1	120.26 (7)
H13B—C13—H13C	109.5	C8—N3—C9	122.05 (7)
C12—C14—H14A	109.5	C1—N3—C9	116.07 (7)
C12—C14—H14B	109.5	C8—N4—C12	120.47 (8)
H14A—C14—H14B	109.5	C22—O—C19	109.17 (8)
C12—C14—H14C	109.5	C22—O—LI	125.84 (8)
H14A—C14—H14C	109.5	C19—O—LI	119.85 (8)
			
N4—C8—C15—C16	−81.54 (11)	C7—C5—N2—LI^i^	36.11 (10)
N3—C8—C15—C16	91.27 (9)	C6—C5—N2—LI^i^	−85.46 (9)
C8—C15—C16—C17	−174.74 (7)	C7—C5—N2—LI	119.48 (10)
C15—C16—C17—C18	172.52 (8)	C6—C5—N2—LI	−2.09 (13)
O—C19—C20—C21	−20.85 (15)	N4—C8—N3—C1	−144.83 (9)
C19—C20—C21—C22	34.32 (15)	C15—C8—N3—C1	41.78 (11)
C20—C21—C22—O	−35.89 (14)	N4—C8—N3—C9	20.09 (13)
N2—C1—N1—C2	−164.04 (8)	C15—C8—N3—C9	−153.30 (8)
N3—C1—N1—C2	16.86 (13)	N1—C1—N3—C8	53.11 (12)
LI—C1—N1—C2	172.01 (10)	N2—C1—N3—C8	−125.98 (9)
N2—C1—N1—LI	23.95 (9)	LI—C1—N3—C8	−30.5 (2)
N3—C1—N1—LI	−155.15 (9)	N1—C1—N3—C9	−112.67 (9)
C3—C2—N1—C1	−123.41 (9)	N2—C1—N3—C9	68.23 (10)
C4—C2—N1—C1	115.74 (10)	LI—C1—N3—C9	163.75 (18)
C3—C2—N1—LI	40.51 (18)	C10—C9—N3—C8	−80.92 (10)
C4—C2—N1—LI	−80.34 (17)	C11—C9—N3—C8	46.94 (11)
N1—C1—N2—C5	−158.57 (8)	C10—C9—N3—C1	84.59 (10)
N3—C1—N2—C5	20.55 (12)	C11—C9—N3—C1	−147.55 (8)
LI—C1—N2—C5	−136.00 (9)	N3—C8—N4—C12	−173.36 (8)
N1—C1—N2—LI^i^	35.43 (13)	C15—C8—N4—C12	−0.89 (14)
N3—C1—N2—LI^i^	−145.45 (9)	C14—C12—N4—C8	120.28 (10)
LI—C1—N2—LI^i^	58.00 (11)	C13—C12—N4—C8	−119.01 (10)
N1—C1—N2—LI	−22.57 (9)	C21—C22—O—C19	23.85 (12)
N3—C1—N2—LI	156.55 (9)	C21—C22—O—LI	178.16 (10)
C7—C5—N2—C1	−132.84 (8)	C20—C19—O—C22	−1.70 (13)
C6—C5—N2—C1	105.59 (9)	C20—C19—O—LI	−157.79 (11)

Symmetry code: (i) −*x*+2, −*y*+1, −*z*.

### (compound_3) [Bis(tetrahydrofuran)lithium]-di-µ-chlorido-{(N,N'-dicyclohexyl-1-butylamidinato-κ2N1,N2)[N,N',N'',N'''-tetracyclohexyl-1-(1-butylamidinato)guanidinato-κ2N1,N2]holmate(III)} Crystal data

**Table:**

[LiHoCl_2_(C_4_H_8_O)_2_(C_17_H_31_N_2_)(C_30_H_53_N_4_)]	*Z* = 2
*M**_r_* = 1120.17	*F*(000) = 1184
Triclinic, *P*1	*D*_x_ = 1.254 Mg m^−^^3^
*a* = 12.909 (3) Å	Mo *K*α radiation, λ = 0.71073 Å
*b* = 15.095 (3) Å	Cell parameters from 29309 reflections
*c* = 16.786 (3) Å	θ = 2.2–29.5°
α = 100.67 (3)°	µ = 1.47 mm^−^^1^
β = 97.20 (3)°	*T* = 153 K
γ = 109.50 (3)°	Prism, yellow
*V* = 2967.5 (12) Å^3^	0.34 × 0.20 × 0.12 × 0.13 (radius) mm

### (compound_3) [Bis(tetrahydrofuran)lithium]-di-µ-chlorido-{(N,N'-dicyclohexyl-1-butylamidinato-κ2N1,N2)[N,N',N'',N'''-tetracyclohexyl-1-(1-butylamidinato)guanidinato-κ2N1,N2]holmate(III)} Data collection

**Table:**

Stoe IPDS 2T diffractometer	12948 independent reflections
Radiation source: fine-focus sealed tube	9625 reflections with *I* > 2σ(*I*)
Detector resolution: 6.67 pixels mm^-1^	*R*_int_ = 0.078
area detector scans	θ_max_ = 27.0°, θ_min_ = 2.3°
Absorption correction: for a sphere (X-AREA and X-RED; Stoe & Cie, 2002)	*h* = −16→16
*T*_min_ = 0.814, *T*_max_ = 0.889	*k* = −18→19
29449 measured reflections	*l* = −21→21

### (compound_3) [Bis(tetrahydrofuran)lithium]-di-µ-chlorido-{(N,N'-dicyclohexyl-1-butylamidinato-κ2N1,N2)[N,N',N'',N'''-tetracyclohexyl-1-(1-butylamidinato)guanidinato-κ2N1,N2]holmate(III)} Refinement

**Table:**

Refinement on *F*^2^	Primary atom site location: heavy-atom method
Least-squares matrix: full	Secondary atom site location: difference Fourier map
*R*[*F*^2^ > 2σ(*F*^2^)] = 0.048	Hydrogen site location: inferred from neighbouring sites
*wR*(*F*^2^) = 0.074	H-atom parameters constrained
*S* = 0.91	*w* = 1/[σ^2^(*F*_o_^2^) + (0.0158*P*)^2^] where *P* = (*F*_o_^2^ + 2*F*_c_^2^)/3
12948 reflections	(Δ/σ)_max_ = 0.001
751 parameters	Δρ_max_ = 0.98 e Å^−^^3^
552 restraints	Δρ_min_ = −1.68 e Å^−^^3^

### (compound_3) [Bis(tetrahydrofuran)lithium]-di-µ-chlorido-{(N,N'-dicyclohexyl-1-butylamidinato-κ2N1,N2)[N,N',N'',N'''-tetracyclohexyl-1-(1-butylamidinato)guanidinato-κ2N1,N2]holmate(III)} Special details

**Table:**

Geometry. All esds (except the esd in the dihedral angle between two l.s. planes) are estimated using the full covariance matrix. The cell esds are taken into account individually in the estimation of esds in distances, angles and torsion angles; correlations between esds in cell parameters are only used when they are defined by crystal symmetry. An approximate (isotropic) treatment of cell esds is used for estimating esds involving l.s. planes.

### (compound_3) [Bis(tetrahydrofuran)lithium]-di-µ-chlorido-{(N,N'-dicyclohexyl-1-butylamidinato-κ2N1,N2)[N,N',N'',N'''-tetracyclohexyl-1-(1-butylamidinato)guanidinato-κ2N1,N2]holmate(III)} Fractional atomic coordinates and isotropic or equivalent isotropic displacement parameters (Å^2^)

**Table:**

	*x*	*y*	*z*	*U*_iso_*/*U*_eq_	Occ. (<1)
HO	0.88596 (2)	0.22098 (2)	0.79652 (2)	0.02381 (5)	
CL1	1.01515 (10)	0.18232 (10)	0.90859 (7)	0.0502 (3)	
CL2	0.92641 (9)	0.38265 (8)	0.90918 (6)	0.0426 (3)	
LI	1.0402 (7)	0.3342 (8)	0.9944 (5)	0.059 (2)	
N1	0.7121 (3)	0.1321 (2)	0.82444 (18)	0.0266 (7)	
N2	0.7857 (3)	0.0572 (2)	0.73204 (19)	0.0319 (8)	
N3	0.6167 (3)	−0.0399 (2)	0.77010 (19)	0.0291 (8)	
N4	0.5748 (3)	−0.2042 (3)	0.7462 (2)	0.0400 (9)	
N5	0.8511 (3)	0.2905 (3)	0.68838 (19)	0.0295 (8)	
N6	1.0136 (3)	0.2673 (3)	0.7108 (2)	0.0315 (8)	
C1	0.7056 (3)	0.0497 (3)	0.7760 (2)	0.0277 (9)	
C2	0.6417 (4)	−0.1197 (3)	0.7857 (3)	0.0324 (10)	
C3	0.7431 (3)	−0.0933 (3)	0.8544 (2)	0.0324 (10)	
H3A	0.797489	−0.028883	0.854679	0.039*	
H3B	0.779768	−0.140929	0.842362	0.039*	
C4	0.7146 (3)	−0.0907 (3)	0.9401 (2)	0.0359 (10)	
H4A	0.669097	−0.049866	0.949777	0.043*	
H4B	0.669505	−0.157029	0.943057	0.043*	
C5	0.8207 (4)	−0.0503 (4)	1.0069 (3)	0.0411 (11)	
H5A	0.867870	0.013966	1.000824	0.049*	
H5B	0.863621	−0.093570	0.998476	0.049*	
C6	0.7985 (4)	−0.0397 (4)	1.0953 (3)	0.0552 (14)	
H6A	0.870064	−0.013388	1.135146	0.083*	
H6B	0.757704	0.004407	1.104798	0.083*	
H6C	0.753450	−0.103239	1.102447	0.083*	
C7	0.6469 (4)	0.1335 (3)	0.8895 (2)	0.0309 (10)	
H7	0.589271	0.067236	0.881307	0.037*	
C8	0.5874 (4)	0.2047 (3)	0.8852 (3)	0.0376 (11)	
H8A	0.536844	0.185592	0.830440	0.045*	
H8B	0.643569	0.270306	0.891481	0.045*	
C9	0.5193 (4)	0.2068 (4)	0.9533 (3)	0.0500 (13)	
H9A	0.484465	0.255824	0.950913	0.060*	
H9B	0.458330	0.142864	0.943519	0.060*	
C10	0.5916 (4)	0.2307 (4)	1.0384 (3)	0.0511 (14)	
H10A	0.646167	0.298083	1.051409	0.061*	
H10B	0.543651	0.225935	1.080279	0.061*	
C11	0.6542 (4)	0.1623 (4)	1.0429 (3)	0.0490 (14)	
H11A	0.600010	0.096001	1.037098	0.059*	
H11B	0.705383	0.182756	1.097580	0.059*	
C12	0.7218 (3)	0.1618 (4)	0.9748 (2)	0.0366 (11)	
H12A	0.779745	0.227098	0.983208	0.044*	
H12B	0.760360	0.115525	0.977993	0.044*	
C13	0.7848 (4)	−0.0221 (3)	0.6659 (3)	0.0354 (10)	
H13	0.713095	−0.078757	0.656913	0.042*	
C14	0.8830 (4)	−0.0521 (4)	0.6889 (3)	0.0423 (11)	
H14A	0.876540	−0.076142	0.739779	0.051*	
H14B	0.953624	0.004912	0.700714	0.051*	
C15	0.8872 (5)	−0.1321 (4)	0.6188 (3)	0.0543 (14)	
H15A	0.953655	−0.148673	0.634712	0.065*	
H15B	0.819270	−0.191067	0.609515	0.065*	
C16	0.8938 (5)	−0.0969 (4)	0.5397 (3)	0.0566 (14)	
H16A	0.965698	−0.041969	0.547613	0.068*	
H16B	0.892221	−0.149708	0.494197	0.068*	
C17	0.7982 (5)	−0.0651 (4)	0.5164 (3)	0.0573 (15)	
H17A	0.726760	−0.121539	0.502910	0.069*	
H17B	0.807019	−0.039528	0.466385	0.069*	
C18	0.7937 (4)	0.0131 (4)	0.5865 (3)	0.0442 (12)	
H18A	0.862233	0.071768	0.596644	0.053*	
H18B	0.728130	0.030596	0.570247	0.053*	
C19A	0.5097 (5)	−0.0672 (5)	0.7090 (4)	0.0331 (14)	0.760 (6)
H19A	0.507199	−0.121037	0.663003	0.040*	0.760 (6)
C20A	0.4073 (5)	−0.1061 (6)	0.7469 (4)	0.0429 (18)	0.760 (6)
H20A	0.405727	−0.054363	0.792001	0.051*	0.760 (6)
H20B	0.411903	−0.160300	0.770710	0.051*	0.760 (6)
C21A	0.3004 (5)	−0.1414 (5)	0.6806 (5)	0.0578 (18)	0.760 (6)
H21A	0.298719	−0.197599	0.638679	0.069*	0.760 (6)
H21B	0.234292	−0.162916	0.706398	0.069*	0.760 (6)
C22A	0.2936 (6)	−0.0619 (6)	0.6385 (5)	0.0556 (19)	0.760 (6)
H22A	0.227323	−0.089060	0.592518	0.067*	0.760 (6)
H22B	0.283341	−0.010198	0.678791	0.067*	0.760 (6)
C23A	0.3971 (5)	−0.0187 (5)	0.6052 (5)	0.0500 (18)	0.760 (6)
H23A	0.392214	0.037082	0.583990	0.060*	0.760 (6)
H23B	0.400267	−0.067578	0.558233	0.060*	0.760 (6)
C24A	0.5048 (5)	0.0152 (4)	0.6710 (4)	0.0402 (14)	0.760 (6)
H24A	0.570868	0.037883	0.645383	0.048*	0.760 (6)
H24B	0.506798	0.070018	0.714643	0.048*	0.760 (6)
C19B	0.5113 (16)	−0.0319 (18)	0.7313 (11)	0.042 (3)	0.240 (6)
H19B	0.521468	0.038259	0.744885	0.050*	0.240 (6)
C20B	0.4137 (16)	−0.085 (2)	0.7678 (14)	0.045 (3)	0.240 (6)
H20C	0.431333	−0.060050	0.828658	0.054*	0.240 (6)
H20D	0.401787	−0.155174	0.755793	0.054*	0.240 (6)
C21B	0.3073 (14)	−0.0722 (16)	0.7312 (11)	0.050 (3)	0.240 (6)
H21C	0.243651	−0.109707	0.753477	0.060*	0.240 (6)
H21D	0.317008	−0.003019	0.747349	0.060*	0.240 (6)
C22B	0.2814 (16)	−0.1062 (19)	0.6379 (12)	0.053 (3)	0.240 (6)
H22C	0.266325	−0.176452	0.621643	0.064*	0.240 (6)
H22D	0.213338	−0.095331	0.615060	0.064*	0.240 (6)
C23B	0.3782 (16)	−0.0521 (18)	0.6030 (14)	0.048 (3)	0.240 (6)
H23C	0.390539	0.017755	0.616784	0.058*	0.240 (6)
H23D	0.360483	−0.075539	0.542001	0.058*	0.240 (6)
C24B	0.4863 (13)	−0.0667 (15)	0.6386 (10)	0.041 (3)	0.240 (6)
H24C	0.475836	−0.136095	0.622681	0.049*	0.240 (6)
H24D	0.549806	−0.029687	0.615859	0.049*	0.240 (6)
C25	0.5914 (4)	−0.2901 (3)	0.7618 (3)	0.0490 (13)	
H25	0.635051	−0.275229	0.819387	0.059*	
C26	0.4763 (5)	−0.3690 (4)	0.7521 (3)	0.0653 (16)	
H26A	0.486433	−0.426639	0.766690	0.078*	
H26B	0.434280	−0.345260	0.790956	0.078*	
C27	0.4085 (4)	−0.3980 (4)	0.6640 (3)	0.0577 (15)	
H27A	0.335785	−0.450564	0.659609	0.069*	
H27B	0.392910	−0.341828	0.650828	0.069*	
C28	0.4717 (4)	−0.4319 (4)	0.6028 (3)	0.0584 (15)	
H28A	0.428556	−0.446053	0.545917	0.070*	
H28B	0.479290	−0.492553	0.611991	0.070*	
C29	0.5868 (4)	−0.3566 (4)	0.6113 (3)	0.0555 (15)	
H29A	0.579015	−0.299086	0.594905	0.067*	
H29B	0.627909	−0.383214	0.573360	0.067*	
C30	0.6541 (4)	−0.3261 (4)	0.6997 (3)	0.0547 (13)	
H30A	0.726971	−0.274007	0.703484	0.066*	
H30B	0.669539	−0.381955	0.713760	0.066*	
C31	0.9532 (3)	0.3082 (3)	0.6710 (2)	0.0312 (10)	
C32	0.9966 (3)	0.3686 (3)	0.6105 (2)	0.0357 (10)	
H32A	1.078453	0.404269	0.629160	0.043*	
H32B	0.960005	0.416616	0.609458	0.043*	
C33	0.9724 (4)	0.3042 (4)	0.5237 (3)	0.0429 (12)	
H33A	0.890802	0.266289	0.506849	0.052*	
H33B	1.011278	0.257846	0.525102	0.052*	
C34	1.0095 (5)	0.3607 (4)	0.4587 (3)	0.0615 (15)	
H34A	0.983018	0.315359	0.403234	0.074*	
H34B	0.973580	0.409324	0.459137	0.074*	
C35	1.1355 (5)	0.4118 (5)	0.4732 (4)	0.080 (2)	
H35A	1.154240	0.446504	0.430002	0.120*	
H35B	1.162139	0.457921	0.527510	0.120*	
H35C	1.171581	0.363907	0.471578	0.120*	
C36	0.7672 (3)	0.3214 (3)	0.6481 (3)	0.0356 (10)	
H36	0.805056	0.372309	0.619663	0.043*	
C37	0.6801 (4)	0.2393 (4)	0.5858 (3)	0.0585 (15)	
H37A	0.646912	0.186625	0.612946	0.070*	
H37B	0.715939	0.214275	0.542683	0.070*	
C38	0.5862 (4)	0.2662 (5)	0.5443 (3)	0.0704 (18)	
H38A	0.617167	0.313861	0.511885	0.085*	
H38B	0.528600	0.207747	0.505750	0.085*	
C39	0.5330 (4)	0.3089 (4)	0.6087 (3)	0.0543 (14)	
H39A	0.476913	0.331023	0.581531	0.065*	
H39B	0.493639	0.258428	0.636125	0.065*	
C40	0.6187 (4)	0.3914 (4)	0.6715 (3)	0.0616 (16)	
H40A	0.582190	0.415206	0.714736	0.074*	
H40B	0.651283	0.444841	0.644881	0.074*	
C41	0.7133 (4)	0.3643 (4)	0.7123 (3)	0.0550 (14)	
H41A	0.770903	0.422789	0.750790	0.066*	
H41B	0.682580	0.316731	0.744805	0.066*	
C42	1.1312 (3)	0.2840 (4)	0.7071 (3)	0.0308 (10)	
H42	1.145267	0.300954	0.653577	0.037*	
C43	1.1522 (3)	0.1916 (3)	0.7092 (3)	0.0365 (11)	
H43A	1.105112	0.140195	0.659777	0.044*	
H43B	1.130249	0.169962	0.758879	0.044*	
C44	1.2756 (4)	0.2061 (4)	0.7110 (3)	0.0479 (13)	
H44A	1.287276	0.145537	0.715685	0.057*	
H44B	1.295429	0.220700	0.658566	0.057*	
C45	1.3510 (3)	0.2875 (4)	0.7826 (3)	0.0464 (12)	
H45A	1.430363	0.297481	0.780586	0.056*	
H45B	1.336410	0.270185	0.835252	0.056*	
C46	1.3312 (3)	0.3804 (4)	0.7796 (3)	0.0473 (12)	
H46A	1.353685	0.401361	0.729874	0.057*	
H46B	1.378416	0.431883	0.828959	0.057*	
C47	1.2085 (3)	0.3668 (3)	0.7774 (3)	0.0376 (10)	
H47A	1.188910	0.354205	0.830431	0.045*	
H47B	1.197492	0.427205	0.771297	0.045*	
O1A	1.2012 (6)	0.3939 (5)	1.0059 (6)	0.068 (2)	0.663 (11)
C48A	1.2625 (9)	0.4994 (8)	1.0228 (10)	0.080 (3)	0.663 (11)
H48A	1.274964	0.519623	0.970858	0.096*	0.663 (11)
H48B	1.220589	0.535503	1.051151	0.096*	0.663 (11)
C49A	1.3690 (8)	0.5161 (8)	1.0764 (8)	0.090 (3)	0.663 (11)
H49A	1.431192	0.567465	1.063643	0.107*	0.663 (11)
H49B	1.365638	0.535823	1.135336	0.107*	0.663 (11)
C50A	1.3853 (8)	0.4236 (8)	1.0590 (9)	0.091 (3)	0.663 (11)
H50A	1.436979	0.424596	1.020053	0.109*	0.663 (11)
H50B	1.418617	0.412419	1.110831	0.109*	0.663 (11)
C51A	1.2766 (6)	0.3465 (6)	1.0228 (7)	0.068 (2)	0.663 (11)
H51A	1.280535	0.304691	0.971227	0.081*	0.663 (11)
H51B	1.252386	0.305921	1.062064	0.081*	0.663 (11)
O1B	1.1907 (11)	0.4215 (12)	1.0355 (11)	0.071 (3)	0.337 (11)
C48B	1.2503 (16)	0.5020 (18)	1.0006 (18)	0.077 (3)	0.337 (11)
H48C	1.235041	0.560929	1.022480	0.093*	0.337 (11)
H48D	1.229113	0.484365	0.939389	0.093*	0.337 (11)
C49B	1.3702 (14)	0.5162 (14)	1.0291 (17)	0.085 (3)	0.337 (11)
H49C	1.400914	0.560276	1.085066	0.102*	0.337 (11)
H49D	1.416696	0.542913	0.990343	0.102*	0.337 (11)
C50B	1.3656 (16)	0.4157 (16)	1.0301 (14)	0.082 (3)	0.337 (11)
H50C	1.352174	0.375145	0.973496	0.098*	0.337 (11)
H50D	1.434669	0.416376	1.063665	0.098*	0.337 (11)
C51B	1.2669 (13)	0.3833 (16)	1.0698 (14)	0.078 (3)	0.337 (11)
H51C	1.233379	0.311731	1.057091	0.093*	0.337 (11)
H51D	1.288978	0.408865	1.130623	0.093*	0.337 (11)
O2A	1.0007 (16)	0.3190 (12)	1.0994 (5)	0.0804 (18)	0.823 (11)
C52A	0.9608 (13)	0.3830 (10)	1.1520 (5)	0.086 (2)	0.823 (11)
H52A	0.883103	0.374213	1.127587	0.103*	0.823 (11)
H52B	1.009542	0.451516	1.159370	0.103*	0.823 (11)
C53A	0.9650 (10)	0.3558 (8)	1.2315 (5)	0.101 (3)	0.823 (11)
H53A	1.022040	0.409209	1.275267	0.121*	0.823 (11)
H53B	0.891206	0.341805	1.247827	0.121*	0.823 (11)
C54A	0.9944 (9)	0.2699 (7)	1.2204 (4)	0.092 (2)	0.823 (11)
H54A	1.066163	0.283501	1.258211	0.110*	0.823 (11)
H54B	0.935406	0.216153	1.233507	0.110*	0.823 (11)
C55A	1.0048 (9)	0.2429 (7)	1.1357 (5)	0.090 (2)	0.823 (11)
H55A	1.076793	0.233467	1.133376	0.108*	0.823 (11)
H55B	0.942657	0.181679	1.105878	0.108*	0.823 (11)
O2B	1.001 (8)	0.329 (6)	1.0966 (19)	0.084 (3)	0.177 (11)
C52B	1.054 (4)	0.291 (3)	1.158 (2)	0.088 (4)	0.177 (11)
H52C	1.130016	0.294988	1.149183	0.106*	0.177 (11)
H52D	1.008073	0.222688	1.154448	0.106*	0.177 (11)
C53B	1.060 (4)	0.355 (3)	1.2372 (17)	0.093 (4)	0.177 (11)
H53C	1.042864	0.316691	1.279167	0.112*	0.177 (11)
H53D	1.136071	0.404992	1.257247	0.112*	0.177 (11)
C54B	0.978 (4)	0.400 (3)	1.224 (2)	0.095 (4)	0.177 (11)
H54C	1.011332	0.470084	1.249695	0.114*	0.177 (11)
H54D	0.912345	0.370614	1.248044	0.114*	0.177 (11)
C55B	0.943 (6)	0.383 (5)	1.134 (2)	0.088 (4)	0.177 (11)
H55C	0.860697	0.347129	1.116540	0.106*	0.177 (11)
H55D	0.961018	0.445725	1.117958	0.106*	0.177 (11)

### (compound_3) [Bis(tetrahydrofuran)lithium]-di-µ-chlorido-{(N,N'-dicyclohexyl-1-butylamidinato-κ2N1,N2)[N,N',N'',N'''-tetracyclohexyl-1-(1-butylamidinato)guanidinato-κ2N1,N2]holmate(III)} Atomic displacement parameters (Å^2^)

**Table:**

	*U*^11^	*U*^22^	*U*^33^	*U*^12^	*U*^13^	*U*^23^
HO	0.02778 (9)	0.02410 (10)	0.02243 (8)	0.01058 (7)	0.00729 (6)	0.00895 (7)
CL1	0.0550 (7)	0.0666 (9)	0.0433 (6)	0.0348 (7)	0.0066 (5)	0.0259 (6)
CL2	0.0553 (7)	0.0321 (7)	0.0357 (6)	0.0152 (6)	0.0048 (5)	0.0012 (5)
LI	0.057 (5)	0.074 (7)	0.040 (5)	0.016 (5)	0.000 (4)	0.018 (5)
N1	0.0327 (18)	0.0240 (19)	0.0251 (17)	0.0121 (15)	0.0102 (14)	0.0051 (15)
N2	0.044 (2)	0.025 (2)	0.0302 (18)	0.0167 (17)	0.0132 (15)	0.0046 (16)
N3	0.0317 (18)	0.0219 (19)	0.0303 (18)	0.0061 (16)	0.0033 (14)	0.0073 (16)
N4	0.053 (2)	0.027 (2)	0.034 (2)	0.0082 (19)	0.0046 (17)	0.0087 (18)
N5	0.0266 (18)	0.039 (2)	0.0319 (18)	0.0179 (16)	0.0086 (14)	0.0160 (17)
N6	0.0255 (17)	0.043 (2)	0.0348 (19)	0.0171 (17)	0.0101 (14)	0.0201 (18)
C1	0.034 (2)	0.025 (2)	0.026 (2)	0.0114 (19)	0.0075 (16)	0.0094 (18)
C2	0.040 (2)	0.026 (3)	0.031 (2)	0.010 (2)	0.0107 (18)	0.008 (2)
C3	0.040 (2)	0.029 (2)	0.031 (2)	0.014 (2)	0.0075 (18)	0.0108 (19)
C4	0.040 (2)	0.034 (3)	0.034 (2)	0.010 (2)	0.0089 (18)	0.015 (2)
C5	0.049 (3)	0.042 (3)	0.036 (2)	0.018 (2)	0.006 (2)	0.015 (2)
C6	0.065 (3)	0.071 (4)	0.030 (2)	0.029 (3)	0.002 (2)	0.010 (3)
C7	0.039 (2)	0.028 (3)	0.029 (2)	0.012 (2)	0.0122 (18)	0.008 (2)
C8	0.040 (3)	0.040 (3)	0.034 (2)	0.018 (2)	0.0116 (19)	0.004 (2)
C9	0.049 (3)	0.065 (4)	0.044 (3)	0.031 (3)	0.019 (2)	0.007 (3)
C10	0.051 (3)	0.062 (4)	0.037 (3)	0.021 (3)	0.021 (2)	−0.002 (3)
C11	0.053 (3)	0.063 (4)	0.026 (2)	0.016 (3)	0.015 (2)	0.006 (2)
C12	0.040 (2)	0.048 (3)	0.026 (2)	0.022 (2)	0.0086 (18)	0.009 (2)
C13	0.045 (3)	0.028 (3)	0.037 (2)	0.017 (2)	0.0156 (19)	0.007 (2)
C14	0.059 (3)	0.045 (3)	0.035 (2)	0.029 (3)	0.021 (2)	0.015 (2)
C15	0.083 (4)	0.052 (3)	0.050 (3)	0.046 (3)	0.029 (3)	0.014 (3)
C16	0.087 (4)	0.058 (4)	0.044 (3)	0.042 (3)	0.034 (3)	0.015 (3)
C17	0.087 (4)	0.067 (4)	0.033 (3)	0.048 (3)	0.020 (2)	0.007 (3)
C18	0.068 (3)	0.044 (3)	0.034 (2)	0.036 (3)	0.016 (2)	0.010 (2)
C19A	0.035 (3)	0.028 (3)	0.034 (3)	0.010 (3)	−0.002 (2)	0.010 (2)
C20A	0.038 (3)	0.041 (4)	0.044 (3)	0.008 (3)	0.005 (3)	0.011 (3)
C21A	0.041 (3)	0.052 (4)	0.067 (4)	0.008 (3)	−0.002 (3)	0.011 (3)
C22A	0.047 (3)	0.040 (4)	0.068 (3)	0.015 (3)	−0.015 (3)	0.006 (3)
C23A	0.051 (3)	0.034 (4)	0.052 (3)	0.009 (3)	−0.013 (3)	0.008 (3)
C24A	0.044 (3)	0.031 (3)	0.041 (3)	0.013 (2)	−0.006 (2)	0.008 (2)
C19B	0.039 (4)	0.036 (5)	0.042 (5)	0.006 (5)	0.003 (4)	0.009 (5)
C20B	0.038 (4)	0.041 (5)	0.047 (5)	0.007 (4)	0.000 (4)	0.009 (5)
C21B	0.038 (4)	0.045 (5)	0.056 (5)	0.009 (4)	−0.001 (4)	0.008 (5)
C22B	0.043 (5)	0.042 (5)	0.062 (5)	0.010 (5)	−0.012 (4)	0.011 (5)
C23B	0.048 (5)	0.036 (5)	0.051 (5)	0.011 (5)	−0.012 (4)	0.011 (5)
C24B	0.043 (4)	0.031 (5)	0.041 (4)	0.006 (4)	−0.002 (4)	0.010 (4)
C25	0.073 (3)	0.026 (3)	0.038 (3)	0.011 (3)	−0.001 (2)	0.009 (2)
C26	0.096 (4)	0.029 (3)	0.051 (3)	−0.001 (3)	0.022 (3)	0.006 (3)
C27	0.062 (3)	0.033 (3)	0.060 (3)	0.001 (3)	0.009 (3)	−0.002 (3)
C28	0.080 (4)	0.037 (3)	0.048 (3)	0.023 (3)	−0.006 (3)	−0.005 (3)
C29	0.073 (4)	0.051 (4)	0.047 (3)	0.031 (3)	0.015 (3)	0.004 (3)
C30	0.069 (3)	0.032 (3)	0.060 (3)	0.019 (3)	0.005 (3)	0.010 (3)
C31	0.033 (2)	0.038 (3)	0.026 (2)	0.015 (2)	0.0110 (17)	0.010 (2)
C32	0.039 (2)	0.038 (3)	0.035 (2)	0.015 (2)	0.0110 (18)	0.015 (2)
C33	0.050 (3)	0.047 (3)	0.033 (2)	0.015 (2)	0.017 (2)	0.011 (2)
C34	0.083 (4)	0.071 (4)	0.038 (3)	0.027 (3)	0.025 (3)	0.023 (3)
C35	0.084 (4)	0.095 (5)	0.065 (4)	0.023 (4)	0.041 (3)	0.032 (4)
C36	0.032 (2)	0.048 (3)	0.037 (2)	0.019 (2)	0.0106 (18)	0.024 (2)
C37	0.050 (3)	0.068 (4)	0.059 (3)	0.036 (3)	0.002 (2)	−0.001 (3)
C38	0.049 (3)	0.096 (5)	0.064 (4)	0.042 (3)	−0.011 (3)	0.001 (3)
C39	0.037 (3)	0.070 (4)	0.064 (3)	0.028 (3)	0.008 (2)	0.022 (3)
C40	0.062 (3)	0.065 (4)	0.071 (4)	0.046 (3)	0.008 (3)	0.009 (3)
C41	0.059 (3)	0.061 (4)	0.049 (3)	0.037 (3)	0.002 (2)	0.001 (3)
C42	0.027 (3)	0.039 (3)	0.030 (2)	0.013 (2)	0.0072 (18)	0.0128 (19)
C43	0.032 (2)	0.032 (3)	0.044 (3)	0.011 (2)	0.0085 (19)	0.007 (2)
C44	0.041 (3)	0.045 (3)	0.060 (3)	0.021 (2)	0.014 (2)	0.006 (3)
C45	0.028 (2)	0.056 (3)	0.051 (3)	0.011 (2)	0.003 (2)	0.013 (3)
C46	0.033 (2)	0.044 (3)	0.054 (3)	0.004 (2)	0.001 (2)	0.010 (3)
C47	0.037 (2)	0.030 (3)	0.044 (3)	0.010 (2)	0.0058 (19)	0.009 (2)
O1A	0.048 (3)	0.049 (3)	0.092 (5)	0.017 (3)	−0.009 (3)	−0.003 (3)
C48A	0.062 (4)	0.057 (4)	0.103 (6)	0.021 (3)	−0.007 (4)	−0.005 (4)
C49A	0.065 (4)	0.073 (4)	0.098 (6)	0.010 (3)	−0.014 (4)	−0.007 (5)
C50A	0.066 (4)	0.072 (4)	0.108 (6)	0.010 (4)	−0.026 (4)	0.018 (5)
C51A	0.054 (4)	0.057 (4)	0.087 (5)	0.026 (3)	−0.007 (4)	0.009 (4)
O1B	0.052 (4)	0.058 (5)	0.092 (6)	0.017 (4)	−0.005 (4)	0.009 (5)
C48B	0.064 (5)	0.058 (5)	0.092 (6)	0.016 (4)	−0.006 (5)	0.001 (5)
C49B	0.064 (5)	0.067 (5)	0.102 (7)	0.016 (4)	−0.012 (5)	0.006 (5)
C50B	0.062 (5)	0.069 (5)	0.095 (7)	0.019 (4)	−0.018 (5)	0.008 (5)
C51B	0.061 (4)	0.061 (5)	0.095 (6)	0.017 (4)	−0.012 (4)	0.010 (5)
O2A	0.135 (3)	0.077 (5)	0.038 (2)	0.044 (3)	0.019 (2)	0.026 (2)
C52A	0.150 (5)	0.071 (4)	0.048 (4)	0.046 (4)	0.026 (4)	0.027 (4)
C53A	0.167 (5)	0.087 (5)	0.055 (4)	0.045 (5)	0.030 (4)	0.028 (4)
C54A	0.148 (5)	0.091 (5)	0.054 (3)	0.050 (4)	0.031 (4)	0.037 (4)
C55A	0.145 (5)	0.083 (5)	0.053 (4)	0.047 (4)	0.009 (4)	0.034 (4)
O2B	0.141 (5)	0.077 (6)	0.045 (5)	0.048 (5)	0.019 (5)	0.029 (5)
C52B	0.144 (6)	0.082 (6)	0.050 (5)	0.049 (5)	0.021 (5)	0.030 (5)
C53B	0.152 (6)	0.085 (6)	0.052 (5)	0.046 (6)	0.025 (5)	0.030 (5)
C54B	0.158 (6)	0.080 (6)	0.053 (6)	0.043 (6)	0.025 (6)	0.029 (6)
C55B	0.149 (6)	0.077 (6)	0.048 (5)	0.046 (5)	0.025 (5)	0.026 (5)

### (compound_3) [Bis(tetrahydrofuran)lithium]-di-µ-chlorido-{(N,N'-dicyclohexyl-1-butylamidinato-κ2N1,N2)[N,N',N'',N'''-tetracyclohexyl-1-(1-butylamidinato)guanidinato-κ2N1,N2]holmate(III)} Geometric parameters (Å, º)

**Table:**

HO—N5	2.327 (3)	C26—C27	1.525 (7)
HO—N6	2.339 (3)	C26—H26A	0.9900
HO—N2	2.341 (4)	C26—H26B	0.9900
HO—N1	2.354 (3)	C27—C28	1.509 (7)
HO—CL1	2.6326 (13)	C27—H27A	0.9900
HO—CL2	2.6453 (15)	C27—H27B	0.9900
HO—C31	2.766 (4)	C28—C29	1.510 (7)
HO—C1	2.770 (4)	C28—H28A	0.9900
HO—LI	3.447 (8)	C28—H28B	0.9900
CL1—LI	2.366 (10)	C29—C30	1.524 (7)
CL2—LI	2.306 (9)	C29—H29A	0.9900
LI—O2B	1.86 (5)	C29—H29B	0.9900
LI—O1B	1.898 (17)	C30—H30A	0.9900
LI—O2A	1.928 (13)	C30—H30B	0.9900
LI—O1A	1.937 (11)	C31—C32	1.523 (6)
N1—C1	1.324 (5)	C32—C33	1.524 (6)
N1—C7	1.461 (5)	C32—H32A	0.9900
N2—C1	1.331 (5)	C32—H32B	0.9900
N2—C13	1.469 (5)	C33—C34	1.533 (6)
N3—C2	1.407 (5)	C33—H33A	0.9900
N3—C1	1.429 (5)	C33—H33B	0.9900
N3—C19B	1.49 (2)	C34—C35	1.514 (7)
N3—C19A	1.496 (6)	C34—H34A	0.9900
N4—C2	1.272 (5)	C34—H34B	0.9900
N4—C25	1.447 (6)	C35—H35A	0.9800
N5—C31	1.336 (5)	C35—H35B	0.9800
N5—C36	1.456 (5)	C35—H35C	0.9800
N6—C31	1.334 (5)	C36—C37	1.479 (6)
N6—C42	1.465 (5)	C36—C41	1.515 (6)
C2—C3	1.518 (6)	C36—H36	1.0000
C3—C4	1.527 (5)	C37—C38	1.530 (6)
C3—H3A	0.9900	C37—H37A	0.9900
C3—H3B	0.9900	C37—H37B	0.9900
C4—C5	1.519 (6)	C38—C39	1.509 (7)
C4—H4A	0.9900	C38—H38A	0.9900
C4—H4B	0.9900	C38—H38B	0.9900
C5—C6	1.536 (6)	C39—C40	1.479 (7)
C5—H5A	0.9900	C39—H39A	0.9900
C5—H5B	0.9900	C39—H39B	0.9900
C6—H6A	0.9800	C40—C41	1.536 (6)
C6—H6B	0.9800	C40—H40A	0.9900
C6—H6C	0.9800	C40—H40B	0.9900
C7—C12	1.522 (6)	C41—H41A	0.9900
C7—C8	1.522 (6)	C41—H41B	0.9900
C7—H7	1.0000	C42—C47	1.509 (6)
C8—C9	1.529 (6)	C42—C43	1.513 (7)
C8—H8A	0.9900	C42—H42	1.0000
C8—H8B	0.9900	C43—C44	1.529 (6)
C9—C10	1.515 (6)	C43—H43A	0.9900
C9—H9A	0.9900	C43—H43B	0.9900
C9—H9B	0.9900	C44—C45	1.504 (6)
C10—C11	1.515 (7)	C44—H44A	0.9900
C10—H10A	0.9900	C44—H44B	0.9900
C10—H10B	0.9900	C45—C46	1.515 (7)
C11—C12	1.523 (5)	C45—H45A	0.9900
C11—H11A	0.9900	C45—H45B	0.9900
C11—H11B	0.9900	C46—C47	1.522 (6)
C12—H12A	0.9900	C46—H46A	0.9900
C12—H12B	0.9900	C46—H46B	0.9900
C13—C14	1.511 (6)	C47—H47A	0.9900
C13—C18	1.526 (6)	C47—H47B	0.9900
C13—H13	1.0000	O1A—C51A	1.418 (9)
C14—C15	1.542 (6)	O1A—C48A	1.477 (11)
C14—H14A	0.9900	C48A—C49A	1.463 (10)
C14—H14B	0.9900	C48A—H48A	0.9900
C15—C16	1.520 (7)	C48A—H48B	0.9900
C15—H15A	0.9900	C49A—C50A	1.462 (11)
C15—H15B	0.9900	C49A—H49A	0.9900
C16—C17	1.500 (7)	C49A—H49B	0.9900
C16—H16A	0.9900	C50A—C51A	1.458 (10)
C16—H16B	0.9900	C50A—H50A	0.9900
C17—C18	1.527 (6)	C50A—H50B	0.9900
C17—H17A	0.9900	C51A—H51A	0.9900
C17—H17B	0.9900	C51A—H51B	0.9900
C18—H18A	0.9900	O1B—C51B	1.411 (14)
C18—H18B	0.9900	O1B—C48B	1.471 (15)
C19A—C24A	1.515 (8)	C48B—C49B	1.492 (15)
C19A—C20A	1.525 (7)	C48B—H48C	0.9900
C19A—H19A	1.0000	C48B—H48D	0.9900
C20A—C21A	1.527 (8)	C49B—C50B	1.501 (15)
C20A—H20A	0.9900	C49B—H49C	0.9900
C20A—H20B	0.9900	C49B—H49D	0.9900
C21A—C22A	1.521 (9)	C50B—C51B	1.491 (16)
C21A—H21A	0.9900	C50B—H50C	0.9900
C21A—H21B	0.9900	C50B—H50D	0.9900
C22A—C23A	1.504 (9)	C51B—H51C	0.9900
C22A—H22A	0.9900	C51B—H51D	0.9900
C22A—H22B	0.9900	O2A—C55A	1.409 (12)
C23A—C24A	1.532 (7)	O2A—C52A	1.452 (9)
C23A—H23A	0.9900	C52A—C53A	1.466 (10)
C23A—H23B	0.9900	C52A—H52A	0.9900
C24A—H24A	0.9900	C52A—H52B	0.9900
C24A—H24B	0.9900	C53A—C54A	1.455 (10)
C19B—C24B	1.504 (16)	C53A—H53A	0.9900
C19B—C20B	1.521 (16)	C53A—H53B	0.9900
C19B—H19B	1.0000	C54A—C55A	1.442 (9)
C20B—C21B	1.523 (17)	C54A—H54A	0.9900
C20B—H20C	0.9900	C54A—H54B	0.9900
C20B—H20D	0.9900	C55A—H55A	0.9900
C21B—C22B	1.510 (17)	C55A—H55B	0.9900
C21B—H21C	0.9900	O2B—C55B	1.408 (17)
C21B—H21D	0.9900	O2B—C52B	1.465 (18)
C22B—C23B	1.500 (17)	C52B—C53B	1.473 (17)
C22B—H22C	0.9900	C52B—H52C	0.9900
C22B—H22D	0.9900	C52B—H52D	0.9900
C23B—C24B	1.548 (16)	C53B—C54B	1.455 (17)
C23B—H23C	0.9900	C53B—H53C	0.9900
C23B—H23D	0.9900	C53B—H53D	0.9900
C24B—H24C	0.9900	C54B—C55B	1.464 (17)
C24B—H24D	0.9900	C54B—H54C	0.9900
C25—C30	1.529 (7)	C54B—H54D	0.9900
C25—C26	1.529 (7)	C55B—H55C	0.9900
C25—H25	1.0000	C55B—H55D	0.9900
			
N5—HO—N6	57.33 (11)	C23B—C24B—H24D	109.9
N5—HO—N2	99.94 (12)	H24C—C24B—H24D	108.3
N6—HO—N2	102.32 (12)	N4—C25—C30	110.4 (4)
N5—HO—N1	107.97 (11)	N4—C25—C26	108.4 (4)
N6—HO—N1	154.43 (11)	C30—C25—C26	108.9 (4)
N2—HO—N1	57.02 (11)	N4—C25—H25	109.7
N5—HO—CL1	154.44 (8)	C30—C25—H25	109.7
N6—HO—CL1	98.60 (8)	C26—C25—H25	109.7
N2—HO—CL1	93.27 (9)	C27—C26—C25	111.8 (4)
N1—HO—CL1	97.59 (9)	C27—C26—H26A	109.3
N5—HO—CL2	92.29 (9)	C25—C26—H26A	109.3
N6—HO—CL2	104.14 (10)	C27—C26—H26B	109.3
N2—HO—CL2	153.43 (8)	C25—C26—H26B	109.3
N1—HO—CL2	96.82 (9)	H26A—C26—H26B	107.9
CL1—HO—CL2	85.19 (5)	C28—C27—C26	110.7 (5)
N5—HO—C31	28.81 (10)	C28—C27—H27A	109.5
N6—HO—C31	28.78 (11)	C26—C27—H27A	109.5
N2—HO—C31	105.55 (12)	C28—C27—H27B	109.5
N1—HO—C31	135.06 (11)	C26—C27—H27B	109.5
CL1—HO—C31	126.18 (9)	H27A—C27—H27B	108.1
CL2—HO—C31	96.54 (10)	C27—C28—C29	111.5 (4)
N5—HO—C1	107.74 (12)	C27—C28—H28A	109.3
N6—HO—C1	130.11 (12)	C29—C28—H28A	109.3
N2—HO—C1	28.64 (11)	C27—C28—H28B	109.3
N1—HO—C1	28.49 (11)	C29—C28—H28B	109.3
CL1—HO—C1	94.33 (9)	H28A—C28—H28B	108.0
CL2—HO—C1	124.91 (9)	C28—C29—C30	111.7 (4)
C31—HO—C1	125.31 (12)	C28—C29—H29A	109.3
N5—HO—LI	128.60 (19)	C30—C29—H29A	109.3
N6—HO—LI	104.43 (17)	C28—C29—H29B	109.3
N2—HO—LI	131.46 (19)	C30—C29—H29B	109.3
N1—HO—LI	100.94 (17)	H29A—C29—H29B	107.9
CL1—HO—LI	43.27 (18)	C29—C30—C25	111.7 (4)
CL2—HO—LI	41.95 (18)	C29—C30—H30A	109.3
C31—HO—LI	117.28 (18)	C25—C30—H30A	109.3
C1—HO—LI	117.41 (18)	C29—C30—H30B	109.3
LI—CL1—HO	87.0 (2)	C25—C30—H30B	109.3
LI—CL2—HO	88.0 (3)	H30A—C30—H30B	107.9
O2B—LI—O1B	97 (3)	N6—C31—N5	113.9 (4)
O2A—LI—O1A	111.1 (7)	N6—C31—C32	122.9 (3)
O2B—LI—CL2	113.7 (17)	N5—C31—C32	123.2 (4)
O1B—LI—CL2	116.8 (7)	N6—C31—HO	57.6 (2)
O2A—LI—CL2	116.6 (6)	N5—C31—HO	57.0 (2)
O1A—LI—CL2	118.3 (5)	C32—C31—HO	172.5 (3)
O2B—LI—CL1	115 (3)	C31—C32—C33	110.7 (4)
O1B—LI—CL1	116.3 (7)	C31—C32—H32A	109.5
O2A—LI—CL1	110.5 (7)	C33—C32—H32A	109.5
O1A—LI—CL1	97.8 (5)	C31—C32—H32B	109.5
CL2—LI—CL1	99.7 (3)	C33—C32—H32B	109.5
O2B—LI—HO	131 (3)	H32A—C32—H32B	108.1
O1B—LI—HO	132.5 (6)	C32—C33—C34	113.6 (4)
O2A—LI—HO	129.7 (7)	C32—C33—H33A	108.8
O1A—LI—HO	116.8 (4)	C34—C33—H33A	108.8
CL2—LI—HO	50.07 (15)	C32—C33—H33B	108.8
CL1—LI—HO	49.70 (15)	C34—C33—H33B	108.8
C1—N1—C7	121.6 (3)	H33A—C33—H33B	107.7
C1—N1—HO	93.5 (2)	C35—C34—C33	113.1 (4)
C7—N1—HO	140.0 (3)	C35—C34—H34A	109.0
C1—N2—C13	123.8 (4)	C33—C34—H34A	109.0
C1—N2—HO	93.9 (2)	C35—C34—H34B	109.0
C13—N2—HO	142.2 (3)	C33—C34—H34B	109.0
C2—N3—C1	119.9 (3)	H34A—C34—H34B	107.8
C2—N3—C19B	131.9 (10)	C34—C35—H35A	109.5
C1—N3—C19B	107.1 (10)	C34—C35—H35B	109.5
C2—N3—C19A	113.8 (4)	H35A—C35—H35B	109.5
C1—N3—C19A	119.0 (4)	C34—C35—H35C	109.5
C2—N4—C25	121.5 (4)	H35A—C35—H35C	109.5
C31—N5—C36	123.8 (3)	H35B—C35—H35C	109.5
C31—N5—HO	94.2 (2)	N5—C36—C37	111.0 (4)
C36—N5—HO	141.7 (2)	N5—C36—C41	109.7 (3)
C31—N6—C42	123.3 (4)	C37—C36—C41	109.8 (4)
C31—N6—HO	93.6 (2)	N5—C36—H36	108.8
C42—N6—HO	140.3 (3)	C37—C36—H36	108.8
N1—C1—N2	115.2 (4)	C41—C36—H36	108.8
N1—C1—N3	121.9 (3)	C36—C37—C38	113.5 (4)
N2—C1—N3	122.9 (4)	C36—C37—H37A	108.9
N1—C1—HO	58.0 (2)	C38—C37—H37A	108.9
N2—C1—HO	57.5 (2)	C36—C37—H37B	108.9
N3—C1—HO	176.1 (3)	C38—C37—H37B	108.9
N4—C2—N3	118.1 (4)	H37A—C37—H37B	107.7
N4—C2—C3	127.5 (4)	C39—C38—C37	110.3 (4)
N3—C2—C3	114.2 (4)	C39—C38—H38A	109.6
C2—C3—C4	113.6 (3)	C37—C38—H38A	109.6
C2—C3—H3A	108.8	C39—C38—H38B	109.6
C4—C3—H3A	108.8	C37—C38—H38B	109.6
C2—C3—H3B	108.8	H38A—C38—H38B	108.1
C4—C3—H3B	108.8	C40—C39—C38	110.9 (4)
H3A—C3—H3B	107.7	C40—C39—H39A	109.5
C5—C4—C3	110.8 (3)	C38—C39—H39A	109.5
C5—C4—H4A	109.5	C40—C39—H39B	109.5
C3—C4—H4A	109.5	C38—C39—H39B	109.5
C5—C4—H4B	109.5	H39A—C39—H39B	108.0
C3—C4—H4B	109.5	C39—C40—C41	112.5 (4)
H4A—C4—H4B	108.1	C39—C40—H40A	109.1
C4—C5—C6	113.7 (4)	C41—C40—H40A	109.1
C4—C5—H5A	108.8	C39—C40—H40B	109.1
C6—C5—H5A	108.8	C41—C40—H40B	109.1
C4—C5—H5B	108.8	H40A—C40—H40B	107.8
C6—C5—H5B	108.8	C36—C41—C40	111.2 (4)
H5A—C5—H5B	107.7	C36—C41—H41A	109.4
C5—C6—H6A	109.5	C40—C41—H41A	109.4
C5—C6—H6B	109.5	C36—C41—H41B	109.4
H6A—C6—H6B	109.5	C40—C41—H41B	109.4
C5—C6—H6C	109.5	H41A—C41—H41B	108.0
H6A—C6—H6C	109.5	N6—C42—C47	110.7 (4)
H6B—C6—H6C	109.5	N6—C42—C43	108.9 (4)
N1—C7—C12	111.1 (3)	C47—C42—C43	111.3 (4)
N1—C7—C8	110.5 (3)	N6—C42—H42	108.6
C12—C7—C8	109.0 (4)	C47—C42—H42	108.6
N1—C7—H7	108.7	C43—C42—H42	108.6
C12—C7—H7	108.7	C42—C43—C44	111.5 (4)
C8—C7—H7	108.7	C42—C43—H43A	109.3
C7—C8—C9	110.8 (4)	C44—C43—H43A	109.3
C7—C8—H8A	109.5	C42—C43—H43B	109.3
C9—C8—H8A	109.5	C44—C43—H43B	109.3
C7—C8—H8B	109.5	H43A—C43—H43B	108.0
C9—C8—H8B	109.5	C45—C44—C43	111.2 (4)
H8A—C8—H8B	108.1	C45—C44—H44A	109.4
C10—C9—C8	111.8 (4)	C43—C44—H44A	109.4
C10—C9—H9A	109.3	C45—C44—H44B	109.4
C8—C9—H9A	109.3	C43—C44—H44B	109.4
C10—C9—H9B	109.3	H44A—C44—H44B	108.0
C8—C9—H9B	109.3	C44—C45—C46	110.8 (4)
H9A—C9—H9B	107.9	C44—C45—H45A	109.5
C11—C10—C9	111.2 (4)	C46—C45—H45A	109.5
C11—C10—H10A	109.4	C44—C45—H45B	109.5
C9—C10—H10A	109.4	C46—C45—H45B	109.5
C11—C10—H10B	109.4	H45A—C45—H45B	108.1
C9—C10—H10B	109.4	C45—C46—C47	111.4 (4)
H10A—C10—H10B	108.0	C45—C46—H46A	109.4
C10—C11—C12	110.7 (4)	C47—C46—H46A	109.4
C10—C11—H11A	109.5	C45—C46—H46B	109.4
C12—C11—H11A	109.5	C47—C46—H46B	109.4
C10—C11—H11B	109.5	H46A—C46—H46B	108.0
C12—C11—H11B	109.5	C42—C47—C46	112.0 (4)
H11A—C11—H11B	108.1	C42—C47—H47A	109.2
C7—C12—C11	111.4 (3)	C46—C47—H47A	109.2
C7—C12—H12A	109.3	C42—C47—H47B	109.2
C11—C12—H12A	109.3	C46—C47—H47B	109.2
C7—C12—H12B	109.3	H47A—C47—H47B	107.9
C11—C12—H12B	109.3	C51A—O1A—C48A	110.3 (6)
H12A—C12—H12B	108.0	C51A—O1A—LI	123.6 (6)
N2—C13—C14	110.4 (3)	C48A—O1A—LI	123.8 (7)
N2—C13—C18	108.7 (4)	C49A—C48A—O1A	104.2 (8)
C14—C13—C18	109.2 (3)	C49A—C48A—H48A	110.9
N2—C13—H13	109.5	O1A—C48A—H48A	110.9
C14—C13—H13	109.5	C49A—C48A—H48B	110.9
C18—C13—H13	109.5	O1A—C48A—H48B	110.9
C13—C14—C15	111.5 (4)	H48A—C48A—H48B	108.9
C13—C14—H14A	109.3	C50A—C49A—C48A	105.5 (8)
C15—C14—H14A	109.3	C50A—C49A—H49A	110.6
C13—C14—H14B	109.3	C48A—C49A—H49A	110.6
C15—C14—H14B	109.3	C50A—C49A—H49B	110.6
H14A—C14—H14B	108.0	C48A—C49A—H49B	110.6
C16—C15—C14	109.7 (4)	H49A—C49A—H49B	108.8
C16—C15—H15A	109.7	C51A—C50A—C49A	108.7 (8)
C14—C15—H15A	109.7	C51A—C50A—H50A	110.0
C16—C15—H15B	109.7	C49A—C50A—H50A	110.0
C14—C15—H15B	109.7	C51A—C50A—H50B	110.0
H15A—C15—H15B	108.2	C49A—C50A—H50B	110.0
C17—C16—C15	111.4 (4)	H50A—C50A—H50B	108.3
C17—C16—H16A	109.4	O1A—C51A—C50A	105.6 (7)
C15—C16—H16A	109.4	O1A—C51A—H51A	110.6
C17—C16—H16B	109.4	C50A—C51A—H51A	110.6
C15—C16—H16B	109.4	O1A—C51A—H51B	110.6
H16A—C16—H16B	108.0	C50A—C51A—H51B	110.6
C16—C17—C18	111.3 (4)	H51A—C51A—H51B	108.7
C16—C17—H17A	109.4	C51B—O1B—C48B	109.9 (12)
C18—C17—H17A	109.4	C51B—O1B—LI	116.5 (12)
C16—C17—H17B	109.4	C48B—O1B—LI	125.7 (14)
C18—C17—H17B	109.4	O1B—C48B—C49B	102.4 (12)
H17A—C17—H17B	108.0	O1B—C48B—H48C	111.3
C13—C18—C17	111.0 (4)	C49B—C48B—H48C	111.3
C13—C18—H18A	109.4	O1B—C48B—H48D	111.3
C17—C18—H18A	109.4	C49B—C48B—H48D	111.3
C13—C18—H18B	109.4	H48C—C48B—H48D	109.2
C17—C18—H18B	109.4	C48B—C49B—C50B	103.2 (13)
H18A—C18—H18B	108.0	C48B—C49B—H49C	111.1
N3—C19A—C24A	112.4 (5)	C50B—C49B—H49C	111.1
N3—C19A—C20A	111.6 (5)	C48B—C49B—H49D	111.1
C24A—C19A—C20A	111.1 (5)	C50B—C49B—H49D	111.1
N3—C19A—H19A	107.2	H49C—C49B—H49D	109.1
C24A—C19A—H19A	107.2	C51B—C50B—C49B	99.5 (13)
C20A—C19A—H19A	107.2	C51B—C50B—H50C	111.9
C19A—C20A—C21A	109.9 (5)	C49B—C50B—H50C	111.9
C19A—C20A—H20A	109.7	C51B—C50B—H50D	111.9
C21A—C20A—H20A	109.7	C49B—C50B—H50D	111.9
C19A—C20A—H20B	109.7	H50C—C50B—H50D	109.6
C21A—C20A—H20B	109.7	O1B—C51B—C50B	104.8 (12)
H20A—C20A—H20B	108.2	O1B—C51B—H51C	110.8
C22A—C21A—C20A	111.4 (6)	C50B—C51B—H51C	110.8
C22A—C21A—H21A	109.3	O1B—C51B—H51D	110.8
C20A—C21A—H21A	109.3	C50B—C51B—H51D	110.8
C22A—C21A—H21B	109.3	H51C—C51B—H51D	108.9
C20A—C21A—H21B	109.3	C55A—O2A—C52A	110.0 (7)
H21A—C21A—H21B	108.0	C55A—O2A—LI	124.7 (7)
C23A—C22A—C21A	112.3 (6)	C52A—O2A—LI	125.3 (9)
C23A—C22A—H22A	109.1	O2A—C52A—C53A	105.7 (8)
C21A—C22A—H22A	109.1	O2A—C52A—H52A	110.6
C23A—C22A—H22B	109.1	C53A—C52A—H52A	110.6
C21A—C22A—H22B	109.1	O2A—C52A—H52B	110.6
H22A—C22A—H22B	107.9	C53A—C52A—H52B	110.6
C22A—C23A—C24A	112.3 (6)	H52A—C52A—H52B	108.7
C22A—C23A—H23A	109.1	C54A—C53A—C52A	107.0 (7)
C24A—C23A—H23A	109.1	C54A—C53A—H53A	110.3
C22A—C23A—H23B	109.1	C52A—C53A—H53A	110.3
C24A—C23A—H23B	109.1	C54A—C53A—H53B	110.3
H23A—C23A—H23B	107.9	C52A—C53A—H53B	110.3
C19A—C24A—C23A	110.1 (5)	H53A—C53A—H53B	108.6
C19A—C24A—H24A	109.6	C55A—C54A—C53A	108.6 (7)
C23A—C24A—H24A	109.6	C55A—C54A—H54A	110.0
C19A—C24A—H24B	109.6	C53A—C54A—H54A	110.0
C23A—C24A—H24B	109.6	C55A—C54A—H54B	110.0
H24A—C24A—H24B	108.2	C53A—C54A—H54B	110.0
N3—C19B—C24B	112.2 (15)	H54A—C54A—H54B	108.4
N3—C19B—C20B	110.5 (15)	O2A—C55A—C54A	106.9 (7)
C24B—C19B—C20B	111.1 (15)	O2A—C55A—H55A	110.3
N3—C19B—H19B	107.6	C54A—C55A—H55A	110.3
C24B—C19B—H19B	107.6	O2A—C55A—H55B	110.3
C20B—C19B—H19B	107.6	C54A—C55A—H55B	110.3
C19B—C20B—C21B	110.2 (16)	H55A—C55A—H55B	108.6
C19B—C20B—H20C	109.6	C55B—O2B—C52B	110.4 (17)
C21B—C20B—H20C	109.6	C55B—O2B—LI	124 (3)
C19B—C20B—H20D	109.6	C52B—O2B—LI	123 (3)
C21B—C20B—H20D	109.6	O2B—C52B—C53B	103.3 (17)
H20C—C20B—H20D	108.1	O2B—C52B—H52C	111.1
C22B—C21B—C20B	110.7 (16)	C53B—C52B—H52C	111.1
C22B—C21B—H21C	109.5	O2B—C52B—H52D	111.1
C20B—C21B—H21C	109.5	C53B—C52B—H52D	111.1
C22B—C21B—H21D	109.5	H52C—C52B—H52D	109.1
C20B—C21B—H21D	109.5	C54B—C53B—C52B	107.6 (17)
H21C—C21B—H21D	108.1	C54B—C53B—H53C	110.2
C23B—C22B—C21B	110.3 (16)	C52B—C53B—H53C	110.2
C23B—C22B—H22C	109.6	C54B—C53B—H53D	110.2
C21B—C22B—H22C	109.6	C52B—C53B—H53D	110.2
C23B—C22B—H22D	109.6	H53C—C53B—H53D	108.5
C21B—C22B—H22D	109.6	C53B—C54B—C55B	107.3 (16)
H22C—C22B—H22D	108.1	C53B—C54B—H54C	110.3
C22B—C23B—C24B	110.8 (16)	C55B—C54B—H54C	110.3
C22B—C23B—H23C	109.5	C53B—C54B—H54D	110.3
C24B—C23B—H23C	109.5	C55B—C54B—H54D	110.3
C22B—C23B—H23D	109.5	H54C—C54B—H54D	108.5
C24B—C23B—H23D	109.5	O2B—C55B—C54B	106.8 (16)
H23C—C23B—H23D	108.1	O2B—C55B—H55C	110.4
C19B—C24B—C23B	108.8 (15)	C54B—C55B—H55C	110.4
C19B—C24B—H24C	109.9	O2B—C55B—H55D	110.4
C23B—C24B—H24C	109.9	C54B—C55B—H55D	110.4
C19B—C24B—H24D	109.9	H55C—C55B—H55D	108.6
			
C7—N1—C1—N2	−166.2 (3)	N4—C25—C30—C29	−63.1 (5)
HO—N1—C1—N2	−6.4 (3)	C26—C25—C30—C29	55.9 (6)
C7—N1—C1—N3	15.7 (6)	C42—N6—C31—N5	−174.3 (4)
HO—N1—C1—N3	175.4 (3)	HO—N6—C31—N5	−9.5 (4)
C7—N1—C1—HO	−159.8 (4)	C42—N6—C31—C32	6.3 (6)
C13—N2—C1—N1	−170.7 (3)	HO—N6—C31—C32	171.1 (4)
HO—N2—C1—N1	6.4 (3)	C42—N6—C31—HO	−164.8 (4)
C13—N2—C1—N3	7.5 (6)	C36—N5—C31—N6	−175.4 (4)
HO—N2—C1—N3	−175.4 (3)	HO—N5—C31—N6	9.6 (4)
C13—N2—C1—HO	−177.1 (4)	C36—N5—C31—C32	4.0 (6)
C2—N3—C1—N1	−124.8 (4)	HO—N5—C31—C32	−171.1 (4)
C19B—N3—C1—N1	65.9 (9)	C36—N5—C31—HO	175.1 (4)
C19A—N3—C1—N1	86.9 (5)	N6—C31—C32—C33	87.0 (5)
C2—N3—C1—N2	57.1 (5)	N5—C31—C32—C33	−92.3 (5)
C19B—N3—C1—N2	−112.1 (8)	C31—C32—C33—C34	177.6 (4)
C19A—N3—C1—N2	−91.1 (5)	C32—C33—C34—C35	65.5 (6)
C25—N4—C2—N3	−176.7 (4)	C31—N5—C36—C37	103.3 (5)
C25—N4—C2—C3	−2.1 (7)	HO—N5—C36—C37	−84.7 (5)
C1—N3—C2—N4	−148.1 (4)	C31—N5—C36—C41	−135.2 (4)
C19B—N3—C2—N4	18.0 (11)	HO—N5—C36—C41	36.8 (6)
C19A—N3—C2—N4	1.7 (6)	N5—C36—C37—C38	177.1 (4)
C1—N3—C2—C3	36.6 (5)	C41—C36—C37—C38	55.6 (6)
C19B—N3—C2—C3	−157.3 (10)	C36—C37—C38—C39	−56.1 (7)
C19A—N3—C2—C3	−173.7 (4)	C37—C38—C39—C40	54.4 (7)
N4—C2—C3—C4	−85.4 (5)	C38—C39—C40—C41	−55.2 (7)
N3—C2—C3—C4	89.3 (4)	N5—C36—C41—C40	−176.1 (4)
C2—C3—C4—C5	−172.0 (4)	C37—C36—C41—C40	−53.9 (6)
C3—C4—C5—C6	176.4 (4)	C39—C40—C41—C36	55.2 (6)
C1—N1—C7—C12	107.3 (4)	C31—N6—C42—C47	92.4 (5)
HO—N1—C7—C12	−40.2 (6)	HO—N6—C42—C47	−63.4 (6)
C1—N1—C7—C8	−131.5 (4)	C31—N6—C42—C43	−145.0 (4)
HO—N1—C7—C8	80.9 (5)	HO—N6—C42—C43	59.3 (6)
N1—C7—C8—C9	−179.8 (4)	N6—C42—C43—C44	−176.4 (3)
C12—C7—C8—C9	−57.5 (5)	C47—C42—C43—C44	−54.1 (5)
C7—C8—C9—C10	56.2 (6)	C42—C43—C44—C45	55.8 (5)
C8—C9—C10—C11	−54.3 (6)	C43—C44—C45—C46	−56.3 (5)
C9—C10—C11—C12	54.6 (6)	C44—C45—C46—C47	55.8 (5)
N1—C7—C12—C11	−179.3 (4)	N6—C42—C47—C46	175.1 (4)
C8—C7—C12—C11	58.7 (5)	C43—C42—C47—C46	53.8 (5)
C10—C11—C12—C7	−57.6 (5)	C45—C46—C47—C42	−54.7 (5)
C1—N2—C13—C14	−116.1 (4)	C51A—O1A—C48A—C49A	−18.8 (13)
HO—N2—C13—C14	68.6 (5)	LI—O1A—C48A—C49A	144.5 (9)
C1—N2—C13—C18	124.1 (4)	O1A—C48A—C49A—C50A	23.9 (15)
HO—N2—C13—C18	−51.1 (6)	C48A—C49A—C50A—C51A	−21.4 (15)
N2—C13—C14—C15	−177.3 (4)	C48A—O1A—C51A—C50A	5.8 (12)
C18—C13—C14—C15	−57.9 (5)	LI—O1A—C51A—C50A	−157.6 (11)
C13—C14—C15—C16	57.4 (6)	C49A—C50A—C51A—O1A	9.7 (14)
C14—C15—C16—C17	−55.8 (6)	O2B—LI—O1B—C51B	75 (2)
C15—C16—C17—C18	56.2 (6)	CL2—LI—O1B—C51B	−164.7 (12)
N2—C13—C18—C17	177.6 (4)	CL1—LI—O1B—C51B	−47.2 (15)
C14—C13—C18—C17	57.1 (5)	HO—LI—O1B—C51B	−105.5 (14)
C16—C17—C18—C13	−56.8 (6)	O2B—LI—O1B—C48B	−140 (3)
C2—N3—C19A—C24A	−159.8 (5)	CL2—LI—O1B—C48B	−19 (2)
C1—N3—C19A—C24A	−9.8 (7)	CL1—LI—O1B—C48B	99 (2)
C2—N3—C19A—C20A	74.6 (6)	HO—LI—O1B—C48B	40 (2)
C1—N3—C19A—C20A	−135.4 (5)	C51B—O1B—C48B—C49B	−8 (3)
N3—C19A—C20A—C21A	−175.1 (6)	LI—O1B—C48B—C49B	−155.8 (17)
C24A—C19A—C20A—C21A	58.7 (8)	O1B—C48B—C49B—C50B	33 (3)
C19A—C20A—C21A—C22A	−55.8 (8)	C48B—C49B—C50B—C51B	−44 (2)
C20A—C21A—C22A—C23A	53.4 (8)	C48B—O1B—C51B—C50B	−20 (2)
C21A—C22A—C23A—C24A	−52.7 (8)	LI—O1B—C51B—C50B	131.1 (18)
N3—C19A—C24A—C23A	176.5 (5)	C49B—C50B—C51B—O1B	39 (2)
C20A—C19A—C24A—C23A	−57.7 (7)	C55A—O2A—C52A—C53A	12.7 (17)
C22A—C23A—C24A—C19A	54.5 (8)	LI—O2A—C52A—C53A	−168.1 (13)
C2—N3—C19B—C24B	−76.8 (18)	O2A—C52A—C53A—C54A	−6.5 (16)
C1—N3—C19B—C24B	90.7 (16)	C52A—C53A—C54A—C55A	−1.6 (14)
C2—N3—C19B—C20B	48 (2)	C52A—O2A—C55A—C54A	−13.8 (16)
C1—N3—C19B—C20B	−144.7 (15)	LI—O2A—C55A—C54A	167.0 (11)
N3—C19B—C20B—C21B	176.9 (17)	C53A—C54A—C55A—O2A	9.4 (14)
C24B—C19B—C20B—C21B	−58 (3)	O1B—LI—O2B—C55B	99 (7)
C19B—C20B—C21B—C22B	57 (3)	CL2—LI—O2B—C55B	−25 (9)
C20B—C21B—C22B—C23B	−58 (3)	CL1—LI—O2B—C55B	−138 (7)
C21B—C22B—C23B—C24B	59 (3)	HO—LI—O2B—C55B	−81 (8)
N3—C19B—C24B—C23B	−177.8 (16)	O1B—LI—O2B—C52B	−63 (7)
C20B—C19B—C24B—C23B	58 (2)	CL2—LI—O2B—C52B	174 (6)
C22B—C23B—C24B—C19B	−59 (2)	CL1—LI—O2B—C52B	60 (8)
C2—N4—C25—C30	−95.9 (5)	HO—LI—O2B—C52B	117 (6)
C2—N4—C25—C26	144.9 (4)	C55B—O2B—C52B—C53B	−21 (8)
N4—C25—C26—C27	63.1 (6)	LI—O2B—C52B—C53B	143 (6)
C30—C25—C26—C27	−57.1 (6)	O2B—C52B—C53B—C54B	21 (6)
C25—C26—C27—C28	57.3 (6)	C52B—C53B—C54B—C55B	−14 (6)
C26—C27—C28—C29	−55.2 (6)	C52B—O2B—C55B—C54B	13 (9)
C27—C28—C29—C30	54.6 (6)	LI—O2B—C55B—C54B	−151 (6)
C28—C29—C30—C25	−55.5 (6)	C53B—C54B—C55B—O2B	1 (8)
